# Self-mediated positive selection of T cells sets an obstacle to the recognition of nonself

**DOI:** 10.1073/pnas.2100542118

**Published:** 2021-09-10

**Authors:** Balázs Koncz, Gergő M. Balogh, Benjamin T. Papp, Leó Asztalos, Lajos Kemény, Máté Manczinger

**Affiliations:** ^a^Department of Dermatology and Allergology, University of Szeged, 6720 Szeged, Hungary;; ^b^Szeged Scientists Academy, 6720 Szeged, Hungary;; ^c^Magyar Tudományos Akadémia - Szegedi Tudományegyetem (MTA‐SZTE) Dermatological Research Group, Eötvös Loránd Research Network (ELKH), University of Szeged, 6720 Szeged, Hungary;; ^d^Hungarian Centre of Excellence for Molecular Medicine - University of Szeged (HCEMM-USZ) Skin Research Group, 6720 Szeged, Hungary;; ^e^Biological Research Centre, Institute of Biochemistry, Synthetic and Systems Biology Unit, Eötvös Loránd Research Network (ELKH), 6726 Szeged, Hungary

**Keywords:** adaptive immune recognition, T cell repertoire, infectious diseases, positive selection

## Abstract

Adaptive immune recognition is mediated by the binding of peptide–human leukocyte antigen complexes by T cells. Positive selection of T cells in the thymus is a fundamental step in the generation of a responding T cell repertoire: only those T cells survive that recognize human peptides presented on the surface of cortical thymic epithelial cells. We propose that while this step is essential for optimal immune function, the process results in a defective T cell repertoire because it is mediated by self-peptides. To test our hypothesis, we focused on amino acid motifs of peptides in contact with T cell receptors. We found that motifs rarely or not found in the human proteome are unlikely to be recognized by the immune system just like the ones that are not expressed in cortical thymic epithelial cells or not presented on their surface. Peptides carrying such motifs were especially dissimilar to human proteins. Importantly, we present our main findings on two independent T cell activation datasets and directly demonstrate the absence of naïve T cells in the repertoire of healthy individuals. We also show that T cell cross-reactivity is unable to compensate for the absence of positively selected T cells. Additionally, we show that the proposed mechanism could influence the risk for different infectious diseases. In sum, our results suggest a side effect of T cell positive selection, which could explain the nonresponsiveness to many nonself peptides and could improve the understanding of adaptive immune recognition.

## Significance

It is well established that peptides that are dissimilar to human proteins are more immunogenic. However, the immune system is still unable to recognize a large fraction of highly dissimilar peptides found in a wide variety of pathogens. We propose that this phenomenon could be explained by the mechanism of T cell positive selection. During this process, only those cells survive that recognize human peptides on the surface of thymic epithelial cells. As self-peptides mediate positive selection, the immune system is unable to recognize many nonself peptides, most of which are highly dissimilar to human peptides.

The human immune system has to differentiate between self and nonself. The prerequisite of adaptive immune recognition is the formation of the immunological synapse (1). This structure is made up of human leukocyte antigen (HLA) molecules presenting short peptide sequences to T cells (1). T cell receptors (TCRs) recognize T cell exposed motifs (TCEMs) of peptide sequences (2–5). These are short, usually five amino acid–long motifs in contact with the CDR3 region of TCRs and are not involved in anchoring the peptides to HLA molecules (2–5).

Adaptive immune recognition is dependent on the presence of peptide-specific T cells in the T cell repertoire (6). The T cell repertoire is shaped by positive and negative selection steps in the thymus (6). Positive selection takes place around cortical thymic epithelial cells (cTECs) (6). cTECs present a special set of peptides on the cell surface produced by the thymoproteasome and cathepsin L (6–8). Recognition of these cTEC-specific peptides by T cell precursors (called thymocytes) is essential for the formation of a functioning T cell repertoire. Nonetheless, these peptides are cleavage products of human proteins (6–9). Thymocytes recognizing HLA-bound self-peptides survive, while others die by neglect (7, 9). Positively selected T cells then go through negative selection: T cells binding self-peptide–HLA complexes with high affinity are deleted from the repertoire, referred to as central tolerance (6).

The positive selection of T cells is an essential step in the formation of a responsive T cell repertoire. It has been suggested that both the CD4+ and CD8+ T cell repertoires are skewed to greater self-reactivity and that T cells that bind self-peptides stronger also bind the foreign agonist peptides more effectively (9–11). In other words, self-peptides mediating positive selection can be considered as a “test set” selecting T cells that recognize foreign peptides with higher effectivity. However, is there any negative consequence of this mechanism?

We propose a fundamental side effect of T cell positive selection on the recognition of nonself peptides: as positive selection is mediated by self-peptides, a large fraction of nonself peptides is not recognized by the immune system even if T cells are cross-reactive. To test our hypothesis, we focused on the TCEMs of HLA class I (HLA-I) restricted peptides. As T cell positive selection is mediated by TCEMs of self-peptides, we expected that it is less likely to find specific T cells in the repertoire for TCEMs that are 1) very rare or missing from human proteins, 2) not expressed in, or 3) not presented on the surface of cTECs. Accordingly, we expected that peptides carrying such motifs are less immunogenic. We demonstrate the predictions of our hypothesis on two nonoverlapping T cell activation datasets and provide more direct evidence by examining naïve CD8+ T cell repertoires of healthy individuals. Although it is widely accepted that nonself peptides that are highly dissimilar to human proteins are more immunogenic (12–16), we found that the dominantly nonimmunogenic peptides having rare TCEMs were more dissimilar to human proteins than immunogenic ones. Such peptides dominated the proteome of many intracellular pathogens, and their presentation by HLA-I molecules was associated with an increased risk of infectious diseases. Our results suggest that the self-mediated positive selection of T cells generates a “blind spot” in adaptive immune recognition with implications on the susceptibility to infectious diseases.

## Results

### Dataset Assembly.

To test the predictions of our hypothesis, we focused on the immunogenicity of peptides that are presented by HLA-I molecules, and thus, the lack of T cell response cannot be explained by missing antigen presentation. We collected T cell activation data for nonhuman peptides from the Immune Epitope Database (IEDB) (17) and assembled two nonoverlapping datasets using different criteria (*SI Appendix*, Fig. S1 and Dataset S1).

In the first dataset, we predicted the binding of each peptide to the reported HLA allele and excluded the ones whose HLA-binding was not confirmed by prediction. To note, this approach has already been used by previous studies focusing on peptide immunogenicity (5). In the case of the second dataset, we aimed to control for certain confounding factors that could bias the analysis. First, the computational prediction of HLA-binding can be inaccurate especially for certain HLA alleles (18, 19). Second, previous works have suggested that the overrepresentation of highly similar sequences due to collection bias in the IEDB could influence the analysis results (20, 21). Consequently, we kept allele–peptide pairs if the binding of the peptide to the reported HLA allele was also verified empirically and excluded similar sequences using an iterative method (*Methods* and *SI Appendix*, Fig. S1). Importantly, we also excluded peptides having controversial assay results or discordant results for different HLA alleles from both datasets. We defined peptides with exclusively negative assay results as nonimmunogenic and peptides with dominantly positive assay results as immunogenic (for detailed curation and filtering steps, refer to *Methods* and *SI Appendix*, Fig. S1).

The number of immunogenic and nonimmunogenic peptides was 1,093 and 2,287 in the first dataset and 360 and 275 in the second one. Peptides in the second dataset had high diversity and covered the sequence space more homogeneously after excluding similar sequences (*SI Appendix*, Fig. S2). We analyzed the two nonoverlapping datasets in parallel to present our findings on a large number of peptides (dataset 1) and to ensure that they are not confounded by computational prediction or the presence of similar sequences (dataset 2).

### TCEMs Occurring Very Rarely or Missing from Human Proteins Are Less Likely to Be Immunogenic.

The positive selection of T cells is mediated by peptide sequences found in the human proteome. Certain amino acids of presented peptides are buried in the binding pockets of HLA molecules, and only five amino acids are in contact with TCRs (2–5). These sequence motifs mediate the recognition of presented peptides (2, 22, 23) and, consequently, the positive selection of T cells in the thymus. We expected that motifs very rarely or not found in the human proteome are less likely to be immunogenic because specific T cells are potentially missing from the repertoire as their precursors did not survive positive selection. Similarly to previous studies (2, 4, 22), we defined TCEMs as five amino acid–long sequences between the anchoring positions of presented peptides (*Methods*). We then determined their frequency in the reference human proteome. We aimed to use TCEM frequency in human proteins as a proxy of their presentation on the cell surface. The prevalence of TCEMs in human proteins showed a long-tailed distribution: a large fraction of motifs was rarely or not found in the human proteome, but many still reached high frequencies (*SI Appendix*, Fig. S3). Next, we collected data of immunopeptidomics studies (Dataset S2) and found that the frequency of TCEMs in human proteins can accurately predict their occurrence in HLA-I–bound peptides on the cell surface (*SI Appendix*, Fig. S4). Importantly, the analysis suggested that TCEMs found less than four times in human proteins are unlikely to be presented on the cell surface (*SI Appendix*, Fig. S4).

**Fig. 1.**
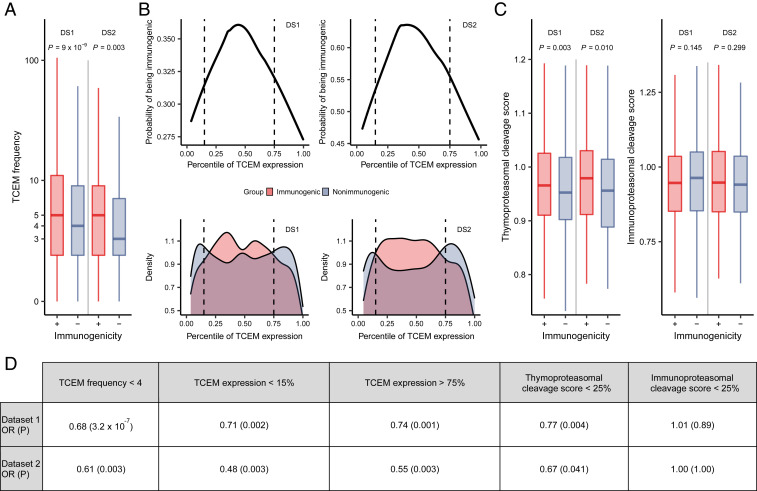
Peptide immunogenicity is influenced by TCEM frequency in human proteins (*A*), TCEM expression in cTECs (*B*), and TCEM presentation on cTECs (*C*). (*A*) The plot indicates the number of times immunogenic (+, *n* = 1,093 and 360 in datasets 1 and 2, respectively) and nonimmunogenic (−, *n* = 2,287 and 275 in datasets 1 and 2, respectively) TCEMs found in human proteins. In both datasets, TCEMs of immunogenic peptides were found more times in human proteins than TCEMs of nonimmunogenic ones. Outliers are not shown for visualization purposes. (*B*) The upper plots show the probability of a TCEM being immunogenic as the function of its expression in cTECs. The curves were fitted using lowess regression (24). The lower plots indicate the probability density of the expression of immunogenic (*n* = 997 and 326 for datasets 1 and 2, respectively) and nonimmunogenic (*n* = 2,040 and 247 for datasets 1 and 2, respectively) TCEMs. For visualization purposes, gene expression values were transformed by calculating their percentile rank. Vertical dashed lines indicate cutoff values used for OR calculation in *D*. (*C*) The likelihood of TCEM formation after thymoproteasomal (*Left*) and immunoproteasomal (*Right*) cleavage is shown. TCEMs of immunogenic peptides were more likely to be generated and presented after thymoproteasomal but not immunoproteasomal cleavage. *n* = 997 and 327 for immunogenic and 2,046 and 248 for nonimmunogenic TCEMs in datasets 1 and 2, respectively. Outliers are not shown for visualization purposes. (*D*) Peptides were classified based on their TCEMs' frequency in human proteins, expression in cTECs, and thymo- or immunoproteasomal cleavage scores. TCEMs found rarely in the human proteome, having low expression in cTECs or low thymoproteasomal cleavage score were less likely to be immunogenic. *P* values of two-sided Fisher’s exact tests are shown. In *A* and *C*, the *P* values of two-sided Wilcoxon’s rank-sum tests are indicated. On *A* and *C*, the bottom and top of boxes indicate the first and third quartile, horizontal lines indicate median, and vertical lines indicate first quartile − 1.5*IQR and third quartile + 1.5*IQR. DS1: dataset 1, DS2: dataset 2.

In line with expectation, nonimmunogenic peptides contained TCEMs that are very uncommon or not found in the human proteome (Fig. 1*A*). Accordingly, motifs occurring less than four times in the human proteome were less likely to be immunogenic than others. (Fig. 1*D*). The result suggests that TCEMs need to occur in sufficient numbers in human proteins to be recognized by the immune system. Otherwise, specific T cells are potentially absent from the repertoire as their precursors have not survived positive selection.

### TCEMs That Are Not Expressed in cTECs Are Less Likely to Be Immunogenic.

In the subsequent analyses, we focused on motifs occurring at least once in the human proteome. It was reported that the HLA-I presentation of peptides is highly dependent on the expression of the encoding gene (25). We assumed that TCEMs encoded by genes having low or undetectable expression in cTECs cannot mediate the positive selection of specific T cells. At the same time, we did not expect an immune response to TCEMs encoded by abundantly expressed housekeeping genes, because the response to these TCEMs may be blocked by central or peripheral immune tolerance (26, 27). In sum, we expected a bimodal relationship between the expression of TCEM-encoding genes and immunogenicity. We downloaded gene expression data of human cTECs from a recent study (28). For each TCEM, we determined the proteins containing its sequence. We then calculated the median expression of genes encoding these proteins to approximate the chance for a given TCEM of being expressed in cTECs. To examine the potentially bimodal relationship between TCEM expression and T cell activation, we plotted the probability for a TCEM of being immunogenic as a function of its expression using lowess smoothing (Fig. 1*B*). We also examined the distribution density of TCEM expression in the immunogenic and nonimmunogenic peptide groups separately (Fig. 1*B*). We found that in line with expectation, TCEMs having either low or high expression in cTECs are similarly less likely to activate T cells than the ones in the medium expression group (Fig. 1 *B* and *D*). These results suggest absent T cell responses to TCEMs that are not expressed at the site of T cell positive selection. As expected, TCEMs in the high expression group were more likely to be found in proteins encoded by housekeeping genes (*SI Appendix*, Fig. S5).

### TCEMs That Are Not Presented on cTECs after Proteasomal Cleavage Are Less Likely to Be Immunogenic.

Even if a given TCEM is expressed in cTECs, proper proteasomal cleavage is essential for its presentation on the cell surface by HLA molecules. Proteasomal cleavage is special in cTECs (6, 8, 29). Thymoproteasomes are exclusively expressed in these cells and are responsible for the generation of peptides that mediate the positive selection of T cells. In contrast with constitutive and immunoproteasomes, thymoproteasomes have a reduced ability to cleave peptide bonds after hydrophobic amino acids (i.e., they have lower chymotrypsin-like activity) (8). A previous study reported the amino acid preference of thymo- and immunoproteasomes around their cleavage sites (8). Using the presented data, we approximated the probability of thymo- and immunoproteasomal cleavage at each position of the reference human proteome. We then calculated a score associated with the chance of a given TCEM being generated after thymoproteasomal cleavage and, thus, presented on the surface of cTECs (*Methods* and *SI Appendix*, Fig. S6). We expected lower immunogenicity for TCEMs that are less likely to be presented on the surface of cTECs after thymoproteasomal cleavage. At the same time, we expected no effect of immunoproteasomal cleavage on immunogenicity because the immunoproteasome has only minor importance in cTECs (8). In line with expectation, TCEMs of immunogenic peptides were more likely to be generated by thymoproteasomal cleavage than nonimmunogenic ones, while immunoproteasomal cleavage did not affect immunogenicity (Fig. 1*C*). Accordingly, TCEMs that are unlikely to be presented on cTECs were less immunogenic (Fig. 1*D*).

### The Robustness of Results.

We reported three lines of evidence suggesting that the positive selection of T cells results in a defective T cell repertoire with implications on the recognition of nonself peptides. First, TCEMs that are very rare or not found in human proteins are less likely to be immunogenic (Fig. 1 *A* and *D*). Second, the scarce expression of TCEMs in cTECs is also associated with lower immunogenicity (Fig. 1 *B* and *D*). Third, TCEMs that are improbably generated by the cTEC-specific thymoproteasome are less likely to be immunogenic (Fig. 1 *C* and *D*).

These effects on immunogenicity held in multivariate logistic regression models, indicating that they are not confounded by and independent of each other (*SI Appendix*, Fig. S7 and Table S1). Similarly, the effect of these attributes was additive: rare TCEMs having low expression in cTECs and low thymoproteasomal cleavage score were less likely to be immunogenic than TCEMs having only one or two of these attributes (*SI Appendix*, Table S2).

Next, we tested whether our findings are confounded by a single amino acid with a peculiar effect on immunogenicity. First, we examined the prevalence of the 20 amino acids in immunogenic and nonimmunogenic TCEMs. The most significant difference was found for tyrosine and phenylalanine enriched in nonimmunogenic motifs and glycine and alanine enriched in immunogenic ones (*SI Appendix*, Table S3). This is in line with expectation as the former amino acids are rarely while the latter ones are commonly found in human proteins (*SI Appendix*, Table S3). Surprisingly, tryptophan, the rarest amino acid, was more common in immunogenic TCEMs, which can be explained by its major role in peptide immunogenicity (30–32). Reassuringly, this phenomenon had no effect on our results as all findings remained significant when we iteratively repeated the analysis by excluding TCEMs containing certain amino acids (*SI Appendix*, Table S3). The hydrophobicity of TCR contact residues is reported to influence peptide immunogenicity (33) and could confound our results. The effect of all TCEM attributes on immunogenicity remained significant when controlling for hydrophobicity in logistic regression models (*SI Appendix*, Fig. S8).

Finally, it is reported that peptides bind to certain HLA variants with secondary anchors in their TCEM region (34). We determined these HLA variants using data from a recent immunopeptidomics study (35) (*Methods*). All of our results held after excluding peptides that bind to all reported HLA variants with secondary anchors at the TCEM region (*n* = 69 and 16 in datasets 1 and 2, respectively; *SI Appendix*, Fig. S9 and Dataset S1).

### The Frequency, Expression, and Presentation of TCEMs Determine the Prevalence of Specific Naïve CD8+ T Cells in the Repertoire.

**Fig. 2.**
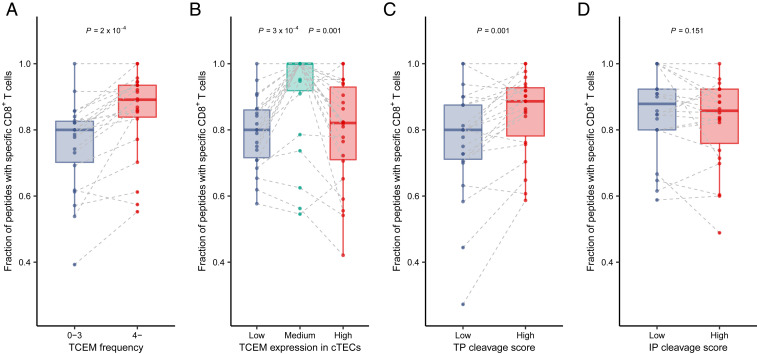
Specific naïve CD8+ T cells were less likely to be present for TCEMs found rarely in human proteins (*A*), having low expression in cTECs (*B*) or low thymoproteasomal cleavage score (*C*), while there was no relationship between immunoproteasomal cleavage score and the prevalence of CD8+ T cells (*D*). The vertical axes represent the fraction of peptides, for which specific T cells were detected. Point pairs (or triplets on *B*) indicate values belonging to a given individual (*n* = 22). Two-sided *P* values of paired Wilcoxon’s rank-sum tests are shown. TCEMs were stratified into expression groups based on tertiles and into thymoproteasomal (TP) or immunoproteasomal (IP) cleavage score groups based on the first quartile. The bottom and top of boxes indicate the first and third quartile, horizontal lines indicate median, and vertical lines indicate first quartile - 1.5*IQR and third quartile + 1.5*IQR.

To confirm our previous findings, we aimed to directly demonstrate the predictions of our hypothesis. Specifically, we expected that it is less likely to find a given naïve T cell in the repertoire that is specific for infrequent TCEMs in human proteins, for TCEMs not expressed in cTECs, or for TCEMs not presented on the surface of cTECs. We used recently published data on the peptide-specificity of naïve CD8+ T cells in the repertoire of healthy individuals (*Methods*) (36). The authors reported the prevalence of naïve CD8+ T cells specific for any of the examined 296 nine amino acid–long (9-mer) SARS-CoV-2 peptides in the repertoire of 27 individuals. In the case of each individual, we focused on sequences that were bound by at least one of their HLA-I alleles (*Methods*), because we expected to find specific T cells for HLA-presented peptides only. We grouped the peptides based on the prevalence, expression, and proteasomal cleavage scores of their TCEMs as described previously for T cell activation datasets. For each individual and in each peptide group, we determined the fraction of HLA-presented peptides that are recognized by at least one TCR in the repertoire. Specific naïve CD8+ T cells were less likely to be present for rare than nonrare TCEMs in the repertoire of healthy individuals (Fig. 2*A*). Similarly, it was less likely to find specific T cells for TCEMs having either negligible or overly high expression in cTECs (Fig. 2*B*). Moreover, TCEMs with low thymoproteasomal cleavage scores were less likely to be associated with the presence of specific T cells in the repertoire (Fig. 2*C*), while the immunoproteasomal cleavage score did not show this relationship (Fig. 2*D*). In sum, our findings for T cell repertoires of healthy individuals confirmed the presented results on the T cell activation datasets.

### Decreased Immunogenicity of Overly Dissimilar Peptides to Human Proteins.

**Fig. 3.**
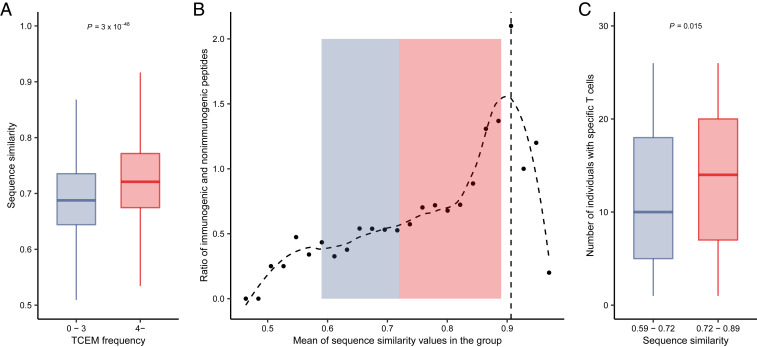
Overly dissimilar peptides to human proteins are less immunogenic. (*A*) Peptides in datasets 1 and 2 with TCEMs found less than four times in human proteins are less similar to the closest hit in the human proteome. (*n* = 1,706 and 2,309 in the 0–3 and 4− TCEM frequency groups, respectively) Outliers are not shown for visualization purposes. (*B*) Peptides in datasets 1 and 2 were pooled and stratified into 25 groups based on similarity. In each group, the ratio between immunogenic and nonimmunogenic peptides was calculated. Groups are shown in increasing order of similarity. The horizontal axis indicates the mean similarity in the given group. The vertical dashed line indicates the group having the highest fraction of immunogenic peptides. The curve was fitted with a cubic smoothing spline method in R (*Methods*). Background shading represents the similarity ranges of peptide groups on *C*. (*C*) T cells specific for overly dissimilar peptides were found in the repertoire of fewer individuals. Peptides were stratified into sequence similarity groups based on the median value (*n* = 149 and 147 in the lower and higher similarity groups, respectively). The similarity ranges are also indicated on *B* with background colors. Note that the dataset of SARS-CoV-2 peptides did not include peptides that are highly similar to human proteins. On *A* and *C*, *P* values of two-sided Wilcoxon’s rank-sum tests are indicated. The bottom and top of boxes indicate the first and third quartile, horizontal lines indicate median, and vertical lines indicate first quartile − 1.5*IQR and third quartile + 1.5*IQR.

Our hypothesis predicted a rather provocative relationship: in contrast with expectation, overly dissimilar peptides are not recognized by the immune system, because self-peptides mediate the positive selection of specific T cells. To test this prediction, for each peptide in the T cell activation datasets, we determined its most similar counterpart in the human proteome using the basic local alignment search tool (BLAST) software as in previous studies (37, 38) (*Methods*). As expected, peptides with very rare TCEMs (occurring zero to three times) in the human proteome had lower similarity to human proteins than other peptides (Fig. 3*A*). Accordingly, overly dissimilar peptides of datasets 1 and 2 were less likely to be immunogenic just like highly similar ones (Fig. 3*B*). Importantly, we found the same relationship when analyzing dissimilarity values of an independent study published recently (37) (*SI Appendix*, Fig. S10). To corroborate these results, we analyzed the self-similarity of SARS-CoV-2 peptides. Reassuringly, we found naïve CD8+ T cells that are specific for highly dissimilar peptides in the repertoire of fewer individuals (Fig. 3*C*).

We conclude that while a given level of peptide dissimilarity to human proteins is essential for self-nonself discrimination, overly dissimilar peptides are less likely to be recognized by the immune system, because specific T cells are not present in the repertoire.

### Cross-Reactivity Is Not Able to Compensate for the Side Effect of Self-Mediated Positive Selection of T Cells.

**Fig. 4.**
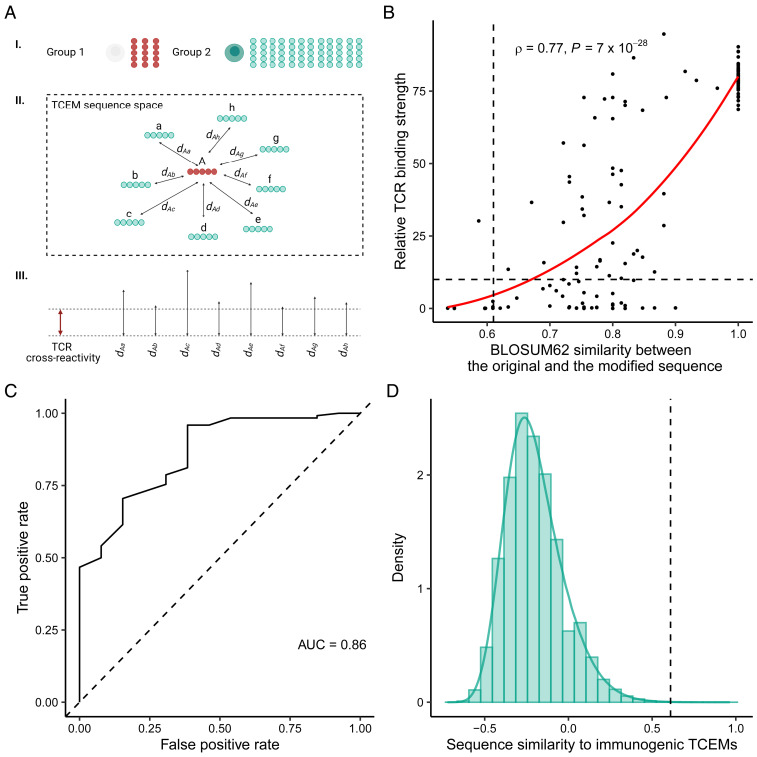
TCR cross-reactivity is not likely to compensate for the defects in the T cell repertoire. (*A*) Schematic diagram of the analysis. To determine whether T cell cross-reactivity is able to bridge defects in the repertoire, we created two groups of TCEMs (I). The first group consisted of motifs, for which we assumed that it is the least likely to find specific positively selected T cells in the repertoire (marked with red color on the figure). The second group consisted of all TCEMs, for which we expected to find specific T cells in the repertoire (marked with green color on the figure). We calculated the pairwise similarity between the members of the two TCEM groups (II). The higher the similarity between two TCEMs, the closer they reside in the sequence space resulting in smaller distance (*d*) values. Next, we estimated the level of T cell cross-reactivity in TCEM sequence space (III). We defined cross-reactivity as the lowest similarity between a given TCEM sequence and the TCR’s cognate TCEM sequence that is needed for a reasonable TCR binding strength and T cell activation. Finally, we determined the number of cases when members of the first and second groups are close enough to be recognized by the same TCR. (*B*) The amino acids of the NY-ESO-1 epitope were sequentially changed, and the binding strength to TCR C^259^ was measured in a previous study (23). The relative binding strength of the modified (*n* = 135) and the original peptide to TCR C^259^ is shown as a function of BLOSUM62 similarity between their TCEM sequences. The horizontal line indicates 10% of the original binding value, which was considered as a cutoff for improbable binding (*Methods*). Spearman’s rho and the *P* value of a two-sided correlation test are indicated. The red line indicates a smooth curve fitted using a cubic smoothing spline method in R (*Methods*). (*C*) The ROC curve demonstrates the accuracy of BLOSUM62 similarity in classifying peptides into binding and nonbinding (i.e., lower than 10% of original binding) groups. AUC: area under the curve. (*D*) The density of all similarity values (*n* = 13,909,210) between TCEMs in group 1 and group 2. Vertical lines on *B* and *D* represent the optimal cutoff (0.61) for classification.

We propose that the mechanism of positive selection results in a defective T cell repertoire. Is the cross-reactivity of TCRs able to compensate for these defects? To answer this question, we first created two groups of TCEMs (Fig. 4*A*). The first group consisted of motifs in datasets 1 and 2, for which we assumed that it is the least likely to find specific positively selected T cells in the repertoire (*n* = 43, they were nonimmunogenic, found less than four times in the human proteome, and had low expression in cTECs and low thymoproteasomal cleavage score; *SI Appendix*, Table S2). The second group consisted of all possible TCEM sequences (*n* = 323,470), for which the presence of specific T cells in the repertoire is likely: they were found more than three times in the human proteome, expressed in cTECs, and had normal thymoproteasomal cleavage score. For each TCEM in the first set, we calculated its BLOSUM62 (blocks substitution matrix 62) similarity to every TCEM in the second set to explain their proximity in sequence space (Fig. 4*A*).

Next, we estimated the level of TCR cross-reactivity in the sequence space of TCEMs. We downloaded empirical data from a recent study. The authors measured the binding strength of the well-known NY-ESO-1 epitope to TCR C^259^, when sequentially replacing every amino acid in different positions of the epitope (23). We determined the BLOSUM62 similarity between the TCEM of the original and the modified peptide sequences and found a strong positive correlation between the similarity of the original to the modified TCEM and the peptide binding strength to TCR C^259^ (Fig. 4*B*).

We then aimed to determine a TCEM similarity cutoff value, under which the binding to the TCR is too weak to induce T cell activation (Fig. 4*A*). To this end, we examined the relationship between the TCR binding strength and the activation of T cells which was reported in the same study. We fixed less than 10% of the original binding strength as insufficient binding because the ability of peptides to activate T cells was negligible below this cutoff (*Methods*). We found that the similarity between the modified and the original TCEM can accurately predict whether the peptide will be bound by the TCR strong enough to cause T cell activation (Fig. 4*C*). We then used an established “cost–benefit” method (39) to determine the optimal TCEM similarity cutoff for binding (*Methods*). We considered this value as the lowest similarity between two TCEM sequences that can be bridged by the examined TCR. Reassuringly, we got a very similar cutoff value, when using data of an independent study on the A6 TCR and its target epitope, the Tax peptide of HTLV-1 (40) (*SI Appendix*, Fig. S11 and *Methods*). As the result of this analysis, we had an estimate on the magnitude of cross-reactivity of a given TCR in sequence space.

We then determined whether T cells that are specific for TCEMs in the second group are able to bind TCEMs in the first group (Fig. 4*D*). We found that only an insignificant minority (ranging from 0.0006 to 0.043% for TCEMs in the first group, median = 0.015%) of similarity values reached the previously determined cutoff values of T cell cross-reactivity (Fig. 4*D*). This result suggests that T cells in the repertoire (specific for TCEMs in the second group) are not likely to recognize TCEMs, whose recognition is negatively affected by self-mediated positive selection (i.e., TCEMs in the first group). Although the result is indicative, it is important to highlight that we inferred cross-reactivity based on the data for two TCRs, and the results need future validation using data of more TCRs.

### Positive Selection of T Cells and Susceptibility to Infections.

**Fig. 5.**
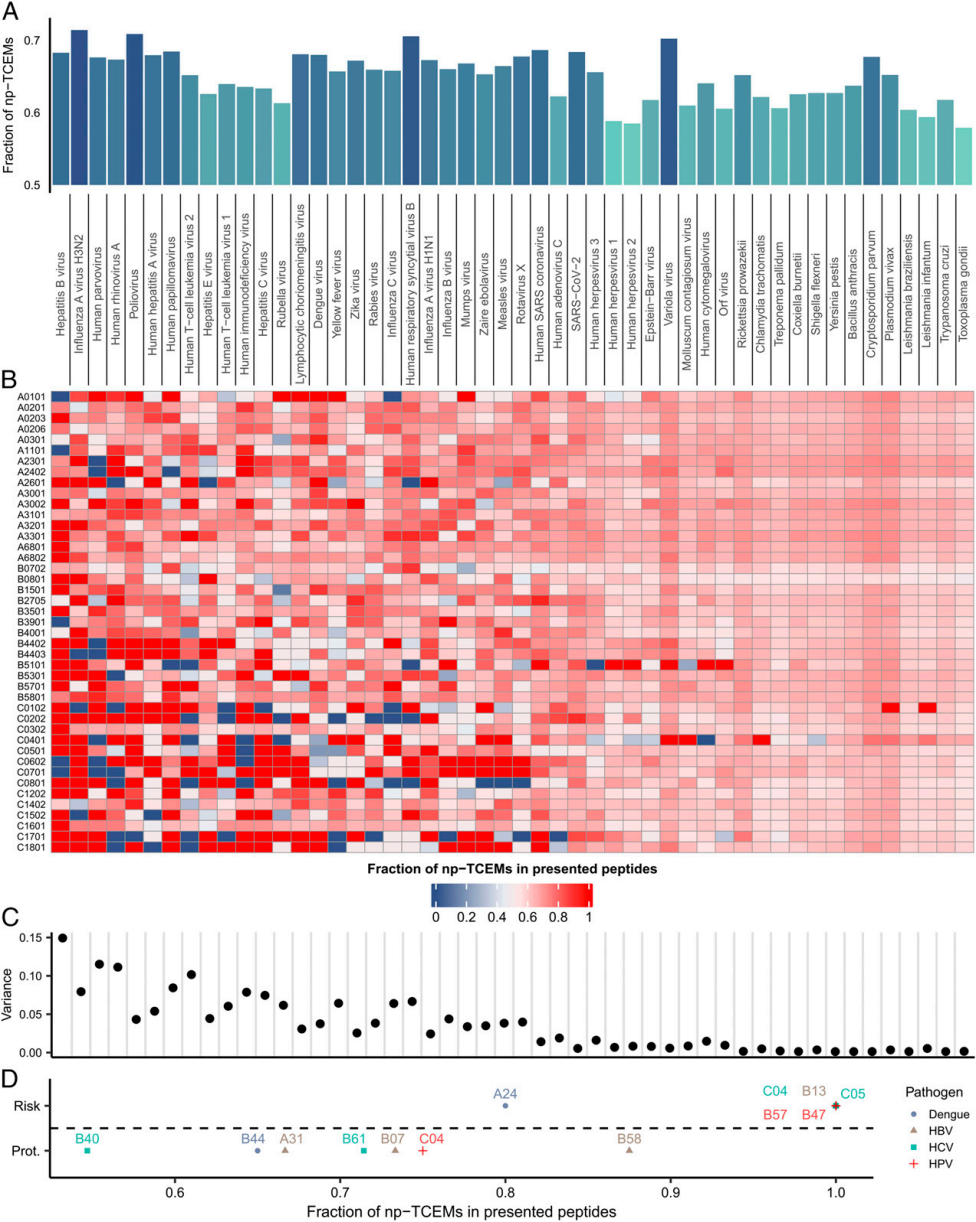
The effect of self-mediated positive selection on the recognition of pathogens. (*A*) The prevalence of np-TCEMs in the proteome of different pathogens (*n* = 50). np-TCEMs were defined as being found less than four times in the human proteome or having low expression in cTECs or low thymoproteasomal cleavage score. Pathogens are ordered by increasing proteome size. (*B*) The heatmap shows the prevalence of np-TCEMs in peptides of intracellular pathogens that are presented by common HLA alleles. (*C*) The plot shows the variance of presented np-TCEMs by different HLA alleles. The variance decreases with increasing proteome size of pathogens (Spearman’s rho: −0.93, two-sided correlation test *P* = 9.13*10^−23^). (*D*) The fraction of np-TCEMs in peptides that are presented by risk (*n* = 6) and protective (*n* = 7) HLA allele groups. Group-specific values were calculated by averaging values for common alleles in each group (*Methods*). In contrast with protective allele groups, predisposing ones present mainly peptides with np-TCEMs in their sequence (two-sided Wilcoxon’s rank-sum test *P* = 0.004). HBV: hepatitis B virus; HCV: hepatitis C virus; and HPV: human papillomavirus. For full pathogen names, refer to Dataset S3.

The adaptive immune recognition of pathogen-associated peptides is essential for the initiation of an effective immune response. Our results suggest that many such peptides are potentially nonimmunogenic, because specific T cells are not found in the repertoire of CD8+ T cells. We aimed to determine the frequency of peptides in proteins of intracellular pathogens that could be affected by the side effect of T cell positive selection to some extent. To this end, we downloaded reference proteomes of 50 common intracellular pathogens. In the proteome of each species, we determined the prevalence of TCEMs that are either rare or not found in human proteins, not or lowly expressed in cTECs, or unlikely to be presented after thymoproteasomal cleavage (we call them np-TCEMs hereafter, referring to TCEMs for which we expect to find specific positively selected T cells with lower probability). We found that the frequency of np-TCEMs is ranging from 58 to 71% in different species. (Fig. 5*A* and Dataset S3).

The high prevalence of np-TCEMs could hinder immune recognition, especially when only a few peptides of the pathogen are presented to T cells. This might be the case when either the proteome of the given pathogen is small and/or the given HLA allele has a narrow binding repertoire. To this end, we predicted the binding of all 9-mer peptides to common HLA-I alleles (*Methods*). For each allele-species pair, we calculated the fraction of np-TCEMs in the presented peptides and visualized the results on a heatmap (Fig. 5*B*). As expected, the fraction of presented peptides with np-TCEMs was highly variable between HLA alleles when the pathogens had small proteomes (Fig. 5*C*). This group of pathogens was dominated by viruses, like Human parvovirus B19, Hepatitis viruses, Human papillomavirus, etc. On the contrary, HLA alleles presented a similar fraction of np-TCEMs from the large proteomes of protozoal and bacterial species (Fig. 5*C*).

We expected that the fraction of HLA-presented np-TCEMs could influence disease risk. To this end, we carried out literature mining to find HLA association meta-analysis data (*Methods*). We found such data for chronic hepatitis B (41), human papillomavirus (42), and dengue virus (43) infection, and Hepatitis C viral persistence after IFN-alpha therapy (44). We selected allele groups with positive or negative associations and determined the prevalence of np-TCEMs in HLA-bound peptides of the causative pathogens. In contrast with protective alleles, risk HLA allele groups bound peptides with dominantly np-TCEMs (Fig. 5*D*).

These results suggest that the proposed side effect of T cell positive selection influences the adaptive immune recognition of intracellular pathogens.

## Discussion

The prevalence of specific T cells in the repertoire is essential for adaptive immune recognition of HLA-presented peptides. It has been suggested that during positive selection, self-peptides on the surface of cTECs can be considered as a test set for thymocytes: cells that recognize these peptides survive and potentially recognize nonself peptides more effectively. Consequently, these cells dominate the immune response to the foreign antigens (9–11). We propose that the nonresponsiveness to many nonself peptides can also be explained by the mechanism of T cell positive selection because it is mediated by self-peptides. In other words, self-mediated positive selection has a negative trade-off on the recognition of foreign peptides. Importantly, our results suggest that T cell cross-reactivity is unable to compensate for this negative consequence of positive selection (Fig. 4).

We presented three lines of evidence supporting our hypothesis on two reliable and nonoverlapping peptide sets (Fig. 1). We focused on the five amino acid–long TCEM region of peptides, because numerous studies suggested that self–nonself discrimination is governed by these short motifs (2–5). Importantly, our analysis on TCR cross-reactivity supports these findings: the TCEM sequences of the modified NY-ESO-1 peptides alone were able to determine the binding of the peptide to the TCR C^259^ (Fig. 4*B*). At the same time, it has been at issue how such short peptides can make it possible for the immune system to differentiate between self and nonself peptides (2, 45). Namely, human peptides contain around 75% of all possible pentamer sequences (2) (73.1% in our analysis) that largely overlap with the ones found in commensal and pathogenic bacteria (2). Our findings suggest that the overlap between self and nonself motifs is far from being disadvantageous; in fact, it is crucial for the positive selection of T cells that are specific for foreign peptides. In other words, the overlap between motifs makes it possible to recognize nonself.

It is an important issue to be clarified whether our findings are affected by regulatory T cell (Treg) activation. Namely, the positivity of T cell assays in our in vitro datasets could reflect the activation of Treg cells to some extent, which could explain the positivity of assays for self-similar peptides. However, peptide immunogenicity in our datasets is supported by dominantly IFN-gamma enzyme-linked immune absorbent spot (ELISpot) assays (Dataset S1). Although a small subset of induced Treg cells is able to produce IFN-gamma (46), clear positivity of IFN-gamma ELISpot assays is predominantly associated with inflammatory but not tolerogenic responses (38). Consequently, it is not likely that our results are confounded by Treg cell activation.

We aimed to support our hypothesis with direct evidence by examining naïve CD8+ T cell repertoires of healthy individuals (Fig. 2). The results suggest that self-mediated positive selection has a negative effect on the prevalence of SARS-CoV-2 peptide-specific T cells in the repertoire (Fig. 2). Importantly, it has already been suggested that “holes” in the T cell repertoire hinder the recognition of certain pathogens (47–50), and the studies explained the presence of such holes by central tolerance (47, 50). On the other hand, Yu et al. suggested that there are no significant holes in the repertoire, because clonal deletion affects only the most self-reactive T cells (51). Consequently, every possible HLA-presented peptide could be recognized by T cells, but many T cells are anergic due to immune tolerance mechanisms. However, our results suggest that the self-dependent positive selection of T cells causes gaps in the immune recognition of nonself peptides potentially through a biased T cell repertoire.

We also estimated the fraction of peptides in pathogens whose recognition could be affected by self-mediated positive selection to some extent. A significant proportion of peptides—varying between 58% and 71% in different species—fell into this category (Fig. 5*A*). If we also consider that around one-third of nonself peptides are indistinguishable from self-ones due to high similarity (52), it is not surprising that at least 50% of HLA-A*02:01–presented vaccinia and HIV sequences were reported to be nonimmunogenic in previous studies (47, 52, 53). At the same time, which peptides are presented to the T cells depends on the specificity of HLA alleles. We showed that HLA alleles that predominantly present peptides whose recognition is potentially hindered by self-mediated positive selection are associated with risk for certain infections (Fig. 5*D*). To note, a similar mechanism could also explain variable responses to vaccines which need further investigation.

**Fig. 6.**
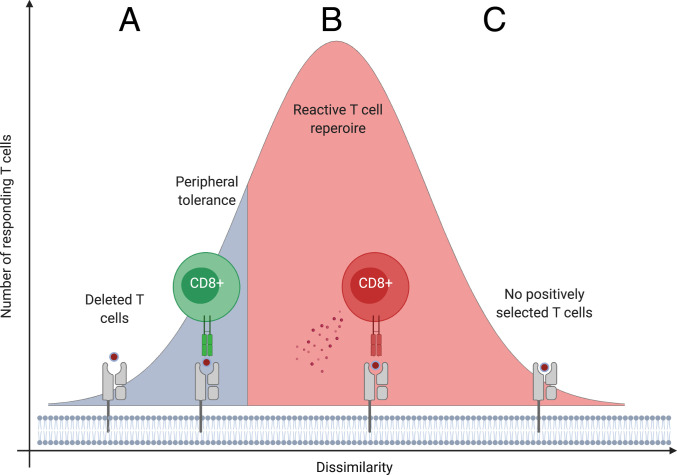
The blindness of immune recognition for peptides that are overly dissimilar to human proteins. (*A*) The immune system tolerates peptides that are similar to self-proteins. T cells recognizing these peptides are either deleted in the thymus or unresponsive due to peripheral tolerance mechanisms (54). (*B*) Peptides with a certain level of dissimilarity to human proteins are recognized as nonself resulting in T cell activation and immune-mediated destruction of cells (Fig. 3*B* and *SI Appendix*, Fig. S10). (*C*) Peptides that are overly dissimilar to human proteins are not recognized by the immune system, because specific positively selected T cells are absent from the repertoire (Fig. 3*B* and *SI Appendix*, Fig. S10).

Finally, our results do not support a common interpretation of self-nonself discrimination, which suggests that the more dissimilar a peptide to self, the more likely it is to be immunogenic (12–15). We showed that the more dissimilar a peptide to human proteins, the less likely it is to find its TCEM in the human proteome (Fig. 3*A*). Consequently, specific positively selected T cells are potentially absent from the repertoire above a level of dissimilarity (Fig. 3 *B* and *C* and *SI Appendix*, Fig. S10). We conclude that although a certain level of dissimilarity is essential for the discrimination of self and nonself, overly dissimilar peptides are potentially not recognized by the immune system (Fig. 6). While our results indicate the importance of this blind spot in the immune response to infections, it is a question to be clarified in future works, whether mutated cancer peptides can also reach this level of dissimilarity. Similarly, testing our hypothesis on HLA-II presented peptides and CD4+ T cells is also an important area of future research.

## Methods

### Collecting and Filtering Peptide Immunogenicity Data.

We collected HLA binding and T cell activation data from the IEDB (17). The IEDB contains experimental data on T and B cell epitopes. Data on MHC binding and T cell specificity are continuously collected from the literature or directly submitted by researchers working in the field. The database is strictly curated and has a standardized decision algorithm to determine whether a given assay is positive or not (55, 56). The authors of the database always refer to experts when they are facing novel assays or immunological content (56). Consequently, the positivity of an assay always means that the interaction between the adaptive immune receptor and the peptide is highly probable (55, 56). We downloaded raw T cell assay results from the website (as of February 3, 2020). We selected nine and 10 amino acid–long linear nonhuman peptides containing only the 20 standard amino acids and tested for HLA-I alleles genotyped with at least 4-digit resolution (*SI Appendix*, Fig. S1). It is important to note that the inclusion of human peptides in our analysis could severely confound our results, because 1) specific positively selected T cells are more likely to be found for these peptides and 2) they are dominantly nonimmunogenic due to central and peripheral tolerance mechanisms (16). Consequently, the presence of human peptides in our datasets could obscure the effect of T cell–positive selection on peptide immunogenicity. Next, we created two independent datasets. The first dataset was created using established methods (5). Specifically, we collected HLA allele–peptide pairs, in which the binding of the peptide by the HLA allele was supported by the prediction results of the NetMHCpan-4.0 algorithm (57) (either the binding affinity was lower than 500 nM, or the binding rank percentile was lower than 2%) (*SI Appendix*, Fig. S1). We considered it particularly important to confirm the HLA-binding of peptides in a unified way using an accurate algorithm, because especially in older studies, HLA restriction of peptides was determined with less accurate computational methods.

To avoid the inaccuracy of computational prediction (18, 19), we created the second dataset using raw MHC binding assay data downloaded from the IEDB (17) (as of February 3, 2020). We collected allele–peptide pairs that were found in both MHC binding and T cell assay data at least twice. We considered a given peptide sequence as being bound by the given HLA allele if more than 60% of binding assays were positive. We also aimed to exclude similar sequences from the second dataset. To this end, we used a previously established iterative method yielding peptides with high sequence diversity (58). Briefly, the k-tuple distance between all peptide sequences was determined in each iteration using Clustal Omega 1.2 (59). Peptide pair(s) with the lowest distance values were determined, and the peptide having the lowest mean distance from all other sequences was excluded. We repeated these iterations until only peptides with at least 0.5 k-tuple distance from all other sequences remained in the dataset (60). This distance value corresponds to a maximum 50% overlap between sequences.

In both datasets, we defined allele–peptide pairs with solely negative T cell assays as nonimmunogenic and the ones with more positive than negative T cell assays as immunogenic. Allele–peptide pairs not meeting these criteria were excluded. Peptide sequences tested for multiple alleles but with the opposite T cell activation results were also excluded (*SI Appendix*, Fig. S1). To avoid any overlap between the two datasets, peptides found in both were only kept in the second one. Using the results of a recent large immunopeptidomics study (35), we identified HLA alleles, to which peptides bind with secondary anchor residues in the TCEM region. We considered a peptide position as an anchoring residue to a given HLA allele if the amino acid entropy at the position of allele-bound peptides was lower than 0.8.

### Calculating TCEM Frequency, Expression, and the Probability of Thymo- and Immunoproteasomal Cleavage.

According to previous studies (2–5), we defined TCEMs of nine amino acid–long peptides as amino acids from positions 4 through 8. For 10 amino acid–long sequences, we defined TCEMs as amino acids from positions 5 through 9 according to sequence logos published in a recent immunopeptidomics study (35). We determined the frequency of TCEM sequences in human proteins as follows. We downloaded the reference human proteome from the UniProt database (61) (Proteome ID: UP000005640; only reviewed sequences are included, downloaded on January, 27th 2020). We decomposed each protein in the proteome to overlapping 9-mers, and for each 9-mer, we determined its TCEM sequence (2–5). We then quantified the incidence of every possible TCEM sequence (*n* = 20^5^) in the human proteome.

To calculate the expression of TCEMs in cTECs, we first downloaded gene expression data of cTECs reported in a recent study (unadjusted counts file under GEO accession GSE127209) (28). We scaled columns of the count matrix using the calcNormFactors function in the edgeR R library (62, 63). Next, we calculated reads per kilobase per million mapped reads (RPKM) values using the rpkm function of edgeR and exon length data of the GenomicFeatures R library (64). Then, for each gene, we determined the median RPKM value in cTEC samples. We matched ENSEMBL gene IDs (used in the expression dataset) with UniProt IDs as follows. Direct conversion between IDs was unsatisfactory, as 40% of UniProt protein IDs in the dataset did not have a corresponding ENSEMBL gene ID in the downloaded expression set. Consequently, we first converted ENSEMBL gene IDs and UniProt IDs to Human Genome Organisation (HUGO) IDs using the org.Hs.eg.db R library and protein information in the UniProt database, respectively. Next, we matched proteins with genes using HUGO IDs. With this approach, we were able to determine the expression of encoding genes for more than 90% of proteins. For each TCEM, we first determined genes encoding proteins that include the given TCEM in their sequence and calculated their median expression. If a given TCEM was found multiple times in the same protein, we included the expression of the encoding gene the same number of times in the calculation. We identified TCEMs encoded by housekeeping genes by using an established list of these genes (65). The relationship between expression and immunogenicity was plotted using lowess regression (24) implemented with the Hmisc R library. The probability density of expression values was plotted after determining the smoothed kernel density estimate by the ggplot2 R library.

The probability of thymo- and immunoproteasomal cleavage was determined using amino acid prevalence data around the cleavage site provided by a previous study (8). Briefly, the authors carried out thymo- and immunoproteasomal digestion of three proteins and determined the fraction of amino acids found at the five positions toward the C and N termini around the cleavage site. They also provided amino acid frequencies in the three proteins that would be found if the proteins were randomly cleaved. We first normalized amino acid prevalence values at each position around the cleavage site by dividing them with their prevalence in the substrates yielding amino acid preference scores: *c*_*i,j*_ referring to the score of amino acid *j* at position *i* around the cleavage site (i ϵ{−5;−4;−3;−2;−1;1;2;3;4;5}) (*SI Appendix*, Fig. S6*A*). Next, we determined the probability of proteasomal cleavage (C) at each site of the human proteome by calculating the median of *c*_*i,j*_ values at positions around the cleavage site (*SI Appendix*, Fig. S6*B*). Then, for each 9-mer peptide in the human proteome, we determined the probability of peptide formation upon proteasomal cleavage by calculating the mean of C values ($\bar{C}$) at the two sites before the N-terminal and after the C-terminal amino acids (*SI Appendix*, Fig. S6*C*). Finally, for each TCEM, we calculated the median of $\bar{C}$ values associated with peptides containing the sequence of the given TCEM (*SI Appendix*, Fig. S6*D*). Note that the presented method was carried out for thymo- and immunoproteasomal cleavage separately.

For each peptide sequence in the study, the most similar sequence in the human proteome was identified using BLAST 2.9.0 (66) with the option of ungapped alignment. The BLOSUM62 similarity between the sequence pairs was calculated as described previously (54).

### Analyzing SARS-CoV-2 TCR Sequencing Dataset.

Data on antigen-specific naïve CD8+ T cells of 27 healthy individuals were downloaded from the website of Adaptive Biotechnologies (36). The authors cocultured naïve CD8+ T cells of healthy donors with dendritic cells, loaded with a pool of all examined peptides of SARS-CoV-2. Then, they used the MIRA technology to identify antigen-specific T cells. The MIRA technology combines conventional T cell assays with immune repertoire sequencing to identify a large number of antigen-specific T cells in the repertoire simultaneously (67). We first determined HLA-I allele–peptide pairs, to which it applies that carrying the given HLA-I allele could potentially be associated with the prevalence of specific naïve CD8+ T cells in the repertoire. Specifically, we predicted the binding of each examined peptide by all HLA alleles carried by any individuals using NetMHCpan-4.0. We associated the prevalence of T cells with carrying a given HLA allele if the predicted binding affinity and rank percentile values suggested strong binding (i.e., affinity was lower than 50 nM, and rank percentile was under 0.5%) and peptide-specific T cells were found in at least two individuals carrying the given allele. We used these strict criteria for HLA-binding to decrease false positive hits. For each individual, we determined the peptides, for which we expected to find specific T cells in the repertoire by considering the previously specified peptide–allele pairs and the HLA genotype of the individual. We included a given individual in the analysis only if we found specific T cells in the repertoire for at least 20 SARS-CoV-2 peptides. This filtering step yielded data on 22 individuals for further analysis.

### Approximating the Level of T Cell Cross-Reactivity.

We acquired data on T cell binding strength reported by two studies (23, 40). Both studies examined the shift in TCR binding strength when sequentially changing amino acids at each epitope position. We narrowed down our analysis to the TCEM sequence and determined the BLOSUM62 similarity between the TCEM of the original and the modified peptides as described previously (54). We determined receiver operating characteristic (ROC) curves and area under the ROC curve (AUC) values using the ROCR R library (68). The optimal cutoff was determined using an established cost–benefit method (39) implemented by the OptimalCutpoints R library (69). In the case of the NY-ESO-1 epitope and TCR C^259^, we were able to determine the level of TCR binding strength under which T cell activation is negligible. We used T cell activation data of the sequentially modified NY-ESO-1 peptides provided by the same study (23). We defined lower than 10% of the original epitope’s TCR binding strength as insufficient binding, because under this value, the median level of T cell activation was only 7.9% of the original.

### Determining TCEMs in Intracellular Pathogens and Analyzing HLA Association Data.

We downloaded the reference proteomes of 50 well-known intracellular pathogens from the UniProt database (61) (on November 18, 2019, SARS-CoV-2 proteome was downloaded on March 26, 2020; Dataset S3). First, we determined the TCEMs of each 9-mer in the proteome of each pathogen. Next, we calculated their prevalence in the human proteome, expression in cTECs, and probability of proteasomal cleavage as explained before. We defined np-TCEMs as the ones found less than four times in the human proteome or having low expression in cTECs or low probability of thymoproteasomal cleavage. We used cutoff values determined for Fig. 2 to define low expression and low probability of thymoproteasomal cleavage. We then predicted the binding of each 9-mer to common HLA alleles with the NetMHCpan-4.0 algorithm (57). We used HLA-A and B alleles listed in a reference set with maximal population coverage found on the IEDB web page (43). As the list did not include data for HLA-C, we selected the first four-digit alleles from all two-digit HLA-C allele classes. To decrease the prevalence of false-positive binding results, we considered a 9-mer to be bound by a given allele if the predicted binding rank percentile value was under 0.5% and the predicted binding affinity value was under 50 nM. To those alleles, for which the prediction could not detect any bound peptides in the proteome of the pathogen, we assigned N peptides having the lowest predicted binding affinity values, in which N refers to the median number of peptides bound by other alleles at the same HLA locus. For each allele–species pair, we calculated the fraction of np-TCEMs in bound peptides and visualized the results on a heatmap.

To identify HLA allele associations with infectious diseases, we carried out literature mining. We aimed to involve only reliable HLA association data in the analysis, so we focused on meta-analyses. We searched PubMed with the “hla infection meta analysis” and “hla association meta analysis” keywords on August 27, 2020. We found HLA association meta-analysis studies for hepatitis B (41), hepatitis C (44), dengue virus (43), and human papillomavirus (HPV) (42). In the case of hepatitis B, C, and HPV studies, we selected significant associations between HLA allele groups and either risk for infections or response to treatment. In the case of dengue virus infection, *P* values were not determined by the authors of the study. They ranked associations of different HLA allele groups with the infection along 17 studies and considered the allele with the best rank as protective ones. We followed the method of the authors and carried out the same analysis but considered only those allele groups that were included in at least 75% of studies to increase the reliability of the analysis. In each study, we calculated the rank percentile of odds ratio values associated with carrying different allele groups. Next, we calculated the average of study-specific rank percentile values for each allele group. We associated the group with the lowest-rank percentile with protection and the group with the highest-rank percentile with susceptibility. As the examined meta-analysis studies were carried out for allele groups or serotypes and not individual alleles, we aggregated the allele-specific values of np-TCEM presentation as follows. For serotypes, we averaged the values specific for the alleles that belong to the given serotype. In the case of allele groups (i.e., HLA type is defined at two-digit resolution), we averaged the values specific for the alleles that are classified as common in the Common and Well-Documented Alleles Catalog (70).

### Statistical Analysis and Visualization.

We used R version 3.6.3 (71) in RStudio version 1.2.5033 environment for statistical analyses. We used the ggplot2 (72), ggpubr, grid, gridExtra, ggsci, scales, png, ComplexHeatmap (73), and ggrepel R libraries for visualization. Smooth curves on plots were fitted with cubic smoothing spline method (74). Figs. 4*A* and 6 were created with BioRender.com.

## Supplementary Material

Supplementary File

Supplementary File

Supplementary File

Supplementary File

## Data Availability

Code data have been deposited in GitHub (https://github.com/KBalazs1987/ImObsRecognition) and Mendeley Data (https://dx.doi.org/10.17632/63xr979845.1). All other study data are included in the article and/or supporting information. Previously published data were used for this work (https://www.iedb.org/ and https://adaptivepublic.blob.core.windows.net/publishedproject-supplements/covid-2020/ImmuneCODE-MIRA-Release002.zip).

## References

[1] M. L. Dustin, The immunological synapse. Cancer Immunol. Res. 2, 1023–1033 (2014). 25367977 10.1158/2326-6066.CIR-14-0161 PMC4692051

[2] R. D. Bremel, E. J. Homan, Extensive T-cell epitope repertoire sharing among human proteome, gastrointestinal microbiome, and pathogenic bacteria: Implications for the definition of self. Front. Immunol. 6, 538 (2015). 26557118 10.3389/fimmu.2015.00538 PMC4617169

[3] M. J. Reddehase, J. B. Rothbard, U. H. Koszinowski, A pentapeptide as minimal antigenic determinant for MHC class I-restricted T lymphocytes. Nature 337, 651–653 (1989). 2465495 10.1038/337651a0

[4] R. D. Bremel, E. J. Homan, Frequency patterns of T-cell exposed amino acid motifs in immunoglobulin heavy chain peptides presented by MHCs. Front. Immunol. 5, 541 (2014). 25389426 10.3389/fimmu.2014.00541 PMC4211557

[5] J. J. A. Calis., Properties of MHC class I presented peptides that enhance immunogenicity. PLoS Comput. Biol. 9, e1003266 (2013). 24204222 10.1371/journal.pcbi.1003266 PMC3808449

[6] L. Klein, B. Kyewski, P. M. Allen, K. A. Hogquist, Positive and negative selection of the T cell repertoire: What thymocytes see (and don’t see). Nat. Rev. Immunol. 14, 377–391 (2014). 24830344 10.1038/nri3667 PMC4757912

[7] K. Takada, K. Kondo, Y. Takahama, Generation of peptides that promote positive selection in the thymus. J. Immunol. 198, 2215–2222 (2017). 28264997 10.4049/jimmunol.1601862

[8] K. Sasaki., Thymoproteasomes produce unique peptide motifs for positive selection of CD8(+) T cells. Nat. Commun. 6, 7484 (2015). 26099460 10.1038/ncomms8484 PMC4557289

[9] N. Vrisekoop, J. P. Monteiro, J. N. Mandl, R. N. Germain, Revisiting thymic positive selection and the mature T cell repertoire for antigen. Immunity 41, 181–190 (2014). 25148022 10.1016/j.immuni.2014.07.007 PMC4152861

[10] J. N. Mandl, J. P. Monteiro, N. Vrisekoop, R. N. Germain, T cell-positive selection uses self-ligand binding strength to optimize repertoire recognition of foreign antigens. Immunity 38, 263–274 (2013). 23290521 10.1016/j.immuni.2012.09.011 PMC3785078

[11] R. B. Fulton., The TCR’s sensitivity to self peptide-MHC dictates the ability of naive CD8(+) T cells to respond to foreign antigens. Nat. Immunol. 16, 107–117 (2015). 25419629 10.1038/ni.3043 PMC4270846

[12] M. Yarmarkovich, J. M. Warrington, A. Farrel, J. M. Maris, Identification of SARS-CoV-2 vaccine epitopes predicted to induce long-term population-scale immunity. Cell Rep Med 1, 100036 (2020). 32835302 10.1016/j.xcrm.2020.100036 PMC7276303

[13] M. Ogishi, H. Yotsuyanagi, The landscape of T cell epitope immunogenicity in sequence space. bioRxiv [Preprint] (2018). 10.1101/155317 (Accessed 1 March 2021). PMC6477061 31057550

[14] P. Lydyard, A. Whelan, M. Fanger, BIOS Instant Notes in Immunology (Taylor & Francis, 2011).

[15] S. K. Mohanty, S. K. Mohanty, K. S. Leela, Textbook of Immunology (JP Medical Ltd, 2013).

[16] Y. Xing, K. A. Hogquist, T-cell tolerance: Central and peripheral. Cold Spring Harb. Perspect. Biol. 4, a006957 (2012). 22661634 10.1101/cshperspect.a006957 PMC3367546

[17] R. Vita., The Immune Epitope Database (IEDB): 2018 update. Nucleic Acids Res. 47, D339–D343 (2019). 30357391 10.1093/nar/gky1006 PMC6324067

[18] S. Paul, ., HLA class I alleles are associated with peptide-binding repertoires of different size, affinity, and immunogenicity. J. Immunol. 191, 5831–5839 (2013). 24190657 10.4049/jimmunol.1302101 PMC3872965

[19] M. Nielsen, M. Andreatta, NetMHCpan-3.0; improved prediction of binding to MHC class I molecules integrating information from multiple receptor and peptide length datasets. Genome Med. 8, 33 (2016). 27029192 10.1186/s13073-016-0288-x PMC4812631

[20] M. Collatz., EpiDope: A deep neural network for linear B-cell epitope prediction. Bioinformatics 37, 448–455 (2021). 32915967 10.1093/bioinformatics/btaa773

[21] S. E. C. Caoili, Expressing redundancy among linear-epitope sequence data based on residue-level physicochemical similarity in the context of antigenic cross-reaction. Adv. Bioinforma. 2016, 1276594 (2016). 10.1155/2016/1276594 PMC4870339 27274725

[22] M. G. Rudolph, R. L. Stanfield, I. A. Wilson, How TCRs bind MHCs, peptides, and coreceptors. Annu. Rev. Immunol. 24, 419–466 (2006). 16551255 10.1146/annurev.immunol.23.021704.115658

[23] A. R. Karapetyan., TCR fingerprinting and off-target peptide identification. Front. Immunol. 10, 2501 (2019). 31695703 10.3389/fimmu.2019.02501 PMC6817589

[24] R. Andersen, Nonparametric methods for modeling nonlinearity in regression analysis. Annu. Rev. Sociol. 35, 67–85 (2009).

[25] H. Pearson., MHC class I-associated peptides derive from selective regions of the human genome. J. Clin. Invest. 126, 4690–4701 (2016). 27841757 10.1172/JCI88590 PMC5127664

[26] D. Malhotra., Tolerance is established in polyclonal CD4(+) T cells by distinct mechanisms, according to self-peptide expression patterns. Nat. Immunol. 17, 187–195 (2016). 26726812 10.1038/ni.3327 PMC4718891

[27] D. von Bubnoff., Antigen-presenting cells and tolerance induction. Allergy 57, 2–8 (2002). 11991283 10.1034/j.1398-9995.2002.01150.x

[28] A. J. Coles., Keratinocyte growth factor impairs human thymic recovery from lymphopenia. JCI Insight 5, e125377 (2019). 10.1172/jci.insight.125377 PMC6629095 31063156

[29] S. Murata., Regulation of CD8+ T cell development by thymus-specific proteasomes. Science 316, 1349–1353 (2007). 17540904 10.1126/science.1141915

[30] M. Zeeshan, K. Tyagi, Y. D. Sharma, CD4+ T cell response correlates with naturally acquired antibodies against Plasmodium vivax tryptophan-rich antigens. Infect. Immun. 83, 2018–2029 (2015). 25733522 10.1128/IAI.03095-14 PMC4399064

[31] J. Schmidt., Prediction of neo-epitope immunogenicity reveals TCR recognition determinants and provides insight into immunoediting. Cell Rep. Med. 2, 100194 (2021). 33665637 10.1016/j.xcrm.2021.100194 PMC7897774

[32] T. P. Riley., Structure based prediction of neoantigen immunogenicity. Front. Immunol. 10, 2047 (2019). 31555277 10.3389/fimmu.2019.02047 PMC6724579

[33] D. Chowell., TCR contact residue hydrophobicity is a hallmark of immunogenic CD8+ T cell epitopes. Proc. Natl. Acad. Sci. U.S.A. 112, E1754–E1762 (2015). 25831525 10.1073/pnas.1500973112 PMC4394253

[34] J. Sidney., Quantitative peptide binding motifs for 19 human and mouse MHC class I molecules derived using positional scanning combinatorial peptide libraries. Immunome Res. 4, 2 (2008). 18221540 10.1186/1745-7580-4-2 PMC2248166

[35] S. Sarkizova., A large peptidome dataset improves HLA class I epitope prediction across most of the human population. Nat. Biotechnol. 38, 199–209 (2020). 31844290 10.1038/s41587-019-0322-9 PMC7008090

[36] T. M. Snyder., Magnitude and dynamics of the T-cell response to SARS-CoV-2 infection at both individual and population levels (infectious diseases (except HIV/AIDS). medRxiv [Preprint] (2020) 10.1101/2020.07.31.20165647 (Accessed 30 September 2020).

[37] L. P. Richman, R. H. Vonderheide, A. J. Rech, Neoantigen dissimilarity to the self-proteome predicts immunogenicity and response to immune checkpoint blockade. Cell Syst. 9, 375–382.e4 (2019). 31606370 10.1016/j.cels.2019.08.009 PMC6813910

[38] S. Carrasco Pro., Microbiota epitope similarity either dampens or enhances the immunogenicity of disease-associated antigenic epitopes. PLoS One 13, e0196551 (2018). 29734356 10.1371/journal.pone.0196551 PMC5937769

[39] C. E. Metz, Basic principles of ROC analysis. Semin. Nucl. Med. 8, 283–298 (1978). 112681 10.1016/s0001-2998(78)80014-2

[40] R. S. Gejman., Identification of the targets of T-cell receptor therapeutic agents and cells by use of a high-throughput genetic platform. Cancer Immunol. Res. 8, 672–684 (2020). 32184297 10.1158/2326-6066.CIR-19-0745 PMC7310334

[41] V. Seshasubramanian, G. Soundararajan, P. Ramasamy, Human leukocyte antigen A, B and Hepatitis B infection outcome: A meta-analysis. Infect. Genet. Evol. 66, 392–398 (2018). 28757339 10.1016/j.meegid.2017.07.027

[42] M. Bhaskaran, G. ArunKumar, A meta-analysis of association of human leukocyte antigens A, B, C, DR and DQ with human papillomavirus 16 infection. Infect. Genet. Evol. 68, 194–202 (2019). 30590170 10.1016/j.meegid.2018.12.026

[43] D. Weiskopf., Comprehensive analysis of dengue virus-specific responses supports an HLA-linked protective role for CD8+ T cells. Proc. Natl. Acad. Sci. U.S.A. 110, E2046–E2053 (2013). 23580623 10.1073/pnas.1305227110 PMC3670335

[44] E. Gauthiez.; Swiss Hepatitis C Cohort Study, A systematic review and meta-analysis of HCV clearance. Liver Int. 37, 1431–1445 (2017). 28261910 10.1111/liv.13401

[45] N. J. Burroughs, R. J. de Boer, C. Keşmir, Discriminating self from nonself with short peptides from large proteomes. Immunogenetics 56, 311–320 (2004). 15322777 10.1007/s00251-004-0691-0

[46] V. Daniel, H. Wang, M. Sadeghi, G. Opelz, Interferon-gamma producing regulatory T cells as a diagnostic and therapeutic tool in organ transplantation. Int. Rev. Immunol. 33, 195–211 (2014). 24266365 10.3109/08830185.2013.845181

[47] S. Frankild, R. J. de Boer, O. Lund, M. Nielsen, C. Kesmir, Amino acid similarity accounts for T cell cross-reactivity and for “holes” in the T cell repertoire. PLoS One 3, e1831 (2008). 18350167 10.1371/journal.pone.0001831 PMC2263130

[48] M. Wölfl., Hepatitis C virus immune escape via exploitation of a hole in the T cell repertoire. J. Immunol. 181, 6435–6446 (2008). 18941234 10.4049/jimmunol.181.9.6435 PMC2742502

[49] E. J. Yager., Age-associated decline in T cell repertoire diversity leads to holes in the repertoire and impaired immunity to influenza virus. J. Exp. Med. 205, 711–723 (2008). 18332179 10.1084/jem.20071140 PMC2275391

[50] D. Vidović, P. Matzinger, Unresponsiveness to a foreign antigen can be caused by self-tolerance. Nature 336, 222–225 (1988). 3143074 10.1038/336222a0

[51] W. Yu., Clonal deletion prunes but does not eliminate self-specific αβ CD8(+) T lymphocytes. Immunity 42, 929–941 (2015). 25992863 10.1016/j.immuni.2015.05.001 PMC4455602

[52] J. J. A. Calis, R. J. de Boer, C. Keşmir, Degenerate T-cell recognition of peptides on MHC molecules creates large holes in the T-cell repertoire. PLOS Comput. Biol. 8, e1002412 (2012). 22396638 10.1371/journal.pcbi.1002412 PMC3291541

[53] M. Rolland., Recognition of HIV-1 peptides by host CTL is related to HIV-1 similarity to human proteins. PLoS One 2, e823 (2007). 17786195 10.1371/journal.pone.0000823 PMC1952107

[54] A. Bresciani., T-cell recognition is shaped by epitope sequence conservation in the host proteome and microbiome. Immunology 148, 34–39 (2016). 26789414 10.1111/imm.12585 PMC4819143

[55] R. Vita, B. Peters, A. Sette, The curation guidelines of the immune epitope database and analysis resource. Cytometry A 73, 1066–1070 (2008). 18688821 10.1002/cyto.a.20585 PMC2597159

[56] W. Fleri., The immune epitope database: How data are entered and retrieved. J. Immunol. Res. 2017, 5974574 (2017). 28634590 10.1155/2017/5974574 PMC5467323

[57] V. Jurtz., NetMHCpan-4.0: Improved peptide-MHC class I interaction predictions integrating eluted ligand and peptide binding affinity data. J. Immunol. 199, 3360–3368 (2017). 28978689 10.4049/jimmunol.1700893 PMC5679736

[58] M. Manczinger., Pathogen diversity drives the evolution of generalist MHC-II alleles in human populations. PLoS Biol. 17, e3000131 (2019). 30703088 10.1371/journal.pbio.3000131 PMC6372212

[59] F. Sievers., Fast, scalable generation of high-quality protein multiple sequence alignments using Clustal Omega. Mol. Syst. Biol. 7, 539 (2011). 21988835 10.1038/msb.2011.75 PMC3261699

[60] K. Yang, L. Zhang, Performance comparison between k-tuple distance and four model-based distances in phylogenetic tree reconstruction. Nucleic Acids Res. 36, e33 (2008). 18296485 10.1093/nar/gkn075 PMC2275138

[61] UniProt Consortium, UniProt: A worldwide hub of protein knowledge. Nucleic Acids Res. 47, D506–D515 (2019). 30395287 10.1093/nar/gky1049 PMC6323992

[62] D. J. McCarthy, Y. Chen, G. K. Smyth, Differential expression analysis of multifactor RNA-Seq experiments with respect to biological variation. Nucleic Acids Res. 40, 4288–4297 (2012). 22287627 10.1093/nar/gks042 PMC3378882

[63] M. D. Robinson, D. J. McCarthy, G. K. Smyth, edgeR: A Bioconductor package for differential expression analysis of digital gene expression data. Bioinformatics 26, 139–140 (2010). 19910308 10.1093/bioinformatics/btp616 PMC2796818

[64] M. Lawrence., Software for computing and annotating genomic ranges. PLOS Comput. Biol. 9, e1003118 (2013). 23950696 10.1371/journal.pcbi.1003118 PMC3738458

[65] E. Eisenberg, E. Y. Levanon, Human housekeeping genes, revisited. Trends Genet. 29, 569–574 (2013). 23810203 10.1016/j.tig.2013.05.010

[66] C. Camacho., BLAST+: Architecture and applications. BMC Bioinformatics 10, 421 (2009). 20003500 10.1186/1471-2105-10-421 PMC2803857

[67] M. Klinger., Multiplex identification of antigen-specific T cell receptors using a combination of immune assays and immune receptor sequencing. PLoS One 10, e0141561 (2015). 26509579 10.1371/journal.pone.0141561 PMC4624875

[68] T. Sing, O. Sander, N. Beerenwinkel, T. Lengauer, ROCR: Visualizing classifier performance in R. Bioinformatics 21, 3940–3941 (2005). 16096348 10.1093/bioinformatics/bti623

[69] M. López-Ratón, M. X. Rodríguez-Álvarez, C. C. Suárez, F. G. Sampedro, OptimalCutpoints: An *R* package for selecting optimal cutpoints in diagnostic tests. J. Stat. Soft. 61, 1–36 (2014).

[70] S. J. Mack., Common and well-documented HLA alleles: 2012 update to the CWD catalogue. Tissue Antigens 81, 194–203 (2013). 23510415 10.1111/tan.12093 PMC3634360

[71] R. Ihaka, R. Gentleman, R: A language for data analysis and graphics. J. Comput. Graph. Stat. 5, 299–314 (1996).

[72] H. Wickham, ggplot2: Elegant Graphics for Data Analysis (Springer-Verlag New York, 2016).

[73] Z. Gu, R. Eils, M. Schlesner, Complex heatmaps reveal patterns and correlations in multidimensional genomic data. Bioinformatics 32, 2847–2849 (2016). 27207943 10.1093/bioinformatics/btw313

[74] C. de Boor, A Practical Guide to Splines (Springer-Verlag New York, 1978).

