# Supplementary material for: Self-mediated positive selection of T cells sets an obstacle to the recognition of nonself
Source: Proc Natl Acad Sci U S A. 2021 Sep 10;118(37):e2100542118. doi: 10.1073/pnas.2100542118 (PMC8449404; doi:10.1073/pnas.2100542118)
Supplement: Supplementary File [file pnas.2100542118.sapp.pdf]

PNAS
www.pnas.org

Supplementary Information for

Self-mediated positive selection of T cells sets an obstacle to the recognition of nonself

Balázs Koncz, Gergő M. Balogh, Benjamin T. Papp, Leó Asztalos, Lajos Kemény, Máté Manczinger

Corresponding author: Máté Manczinger
Email: manczinger.mate@med.u-szeged.hu

This PDF file includes:

Figures S1 to S11
Tables S1 to S3
SI References

Other supplementary materials for this manuscript include the following:

Datasets S1 to S3

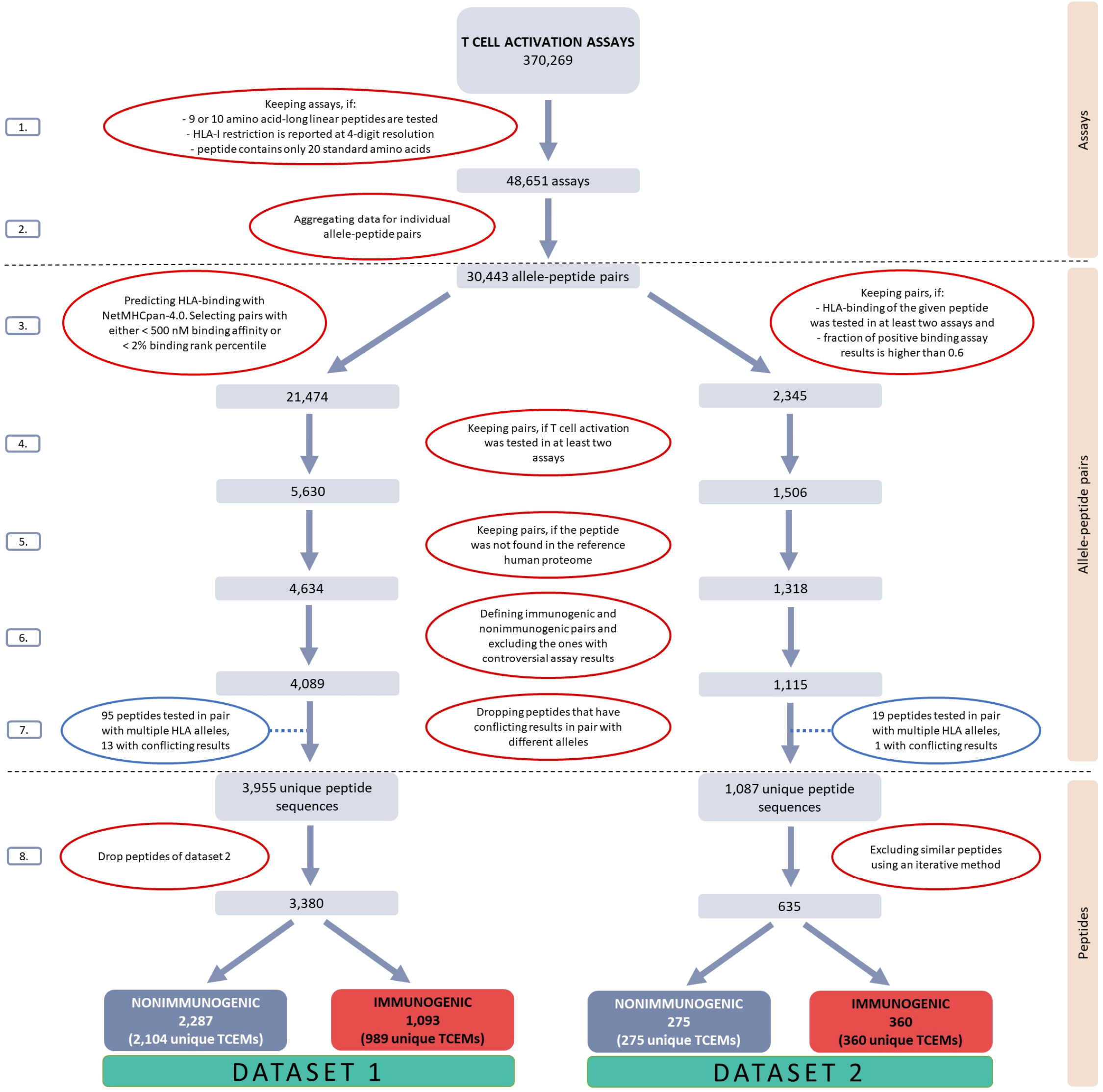

Fig. S1. The assembly of peptide sets used throughout our study. Ellipses indicate filtering criteria at each step. T cell activation data on peptide-HLA pairs were collected from the IEDB and filtered (steps 1 and 2). The HLA binding of peptides was confirmed with computational prediction in the first dataset and experimentally in the second dataset (step 3). Allele-peptide pairs, whose binding was not confirmed were discarded. Also, allele-peptide pairs were excluded, if they were tested in only one T cell assay (step 4) and/or the peptide was found in the reference human proteome (step 5). Next, the allele-peptide pairs were classified into immunogenic and nonimmunogenic groups and the pairs having controversial assay results were excluded (step 6). In both datasets, peptides having conflicting results with different alleles were excluded (step 7). The remaining peptides in dataset 2 were filtered for nonsimilar sequences (step 8). To avoid overlap, peptides found in both datasets were kept only in dataset 2 (step 8).

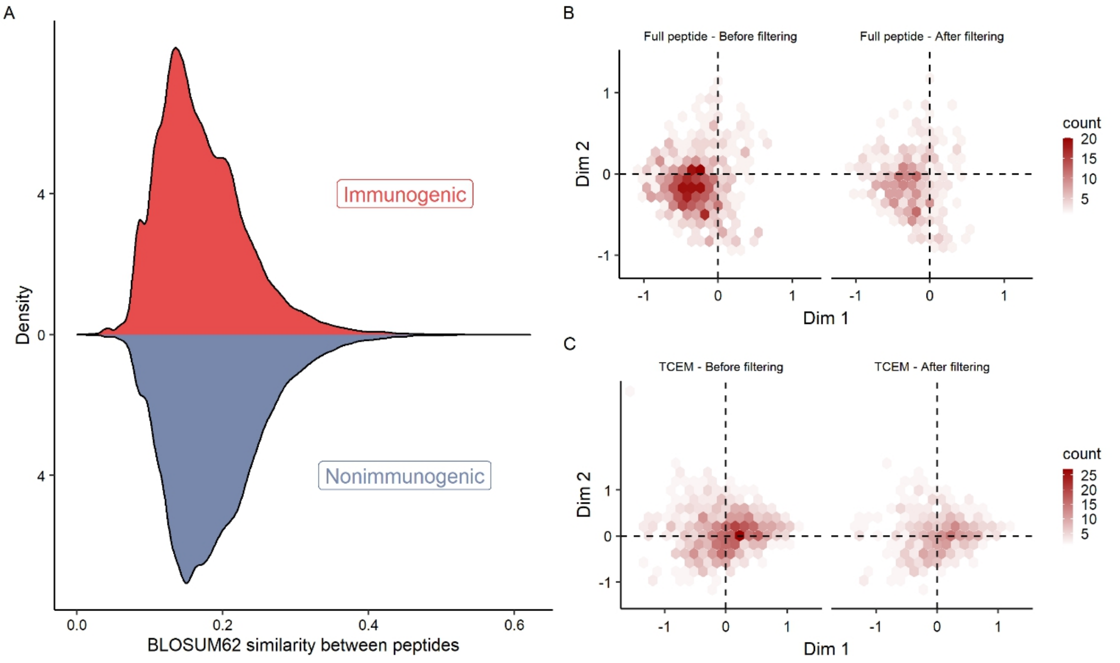

Fig. S2. Diversity of peptide sequences. (*A*) Plots indicate the density of BLOSUM62 similarity values (*n* = 64,620 and 37,675 for immunogenic and nonimmunogenic sequences, respectively) between all pairs of immunogenic and nonimmunogenic peptides in dataset 2. Both groups contain highly diverse and dissimilar sequences. BLOSUM62 similarity values between peptide pairs were calculated with the protr R library (1). (*B*-*C*) Peptide sequences of dataset 2 cover the sequence space more homogeneously after excluding similar sequences (*n* = 853 and 525 before and after filtering). Multiple correspondence analysis (MCA) was carried out on nine amino acid-long peptide sequences as follows. Each position of the peptide (*B*) or its TCEM region (*C*) was treated as a categorical variable having the 20 amino acids as possible categories. The distribution of peptides (*B*) or TCEMs (*C*) in sequence space is shown on MCA biplots. The number of sequences in equal-size hexagons is shown color-coded. The distribution of sequences was less heterogeneous after similarity reduction (*B* and *C*).

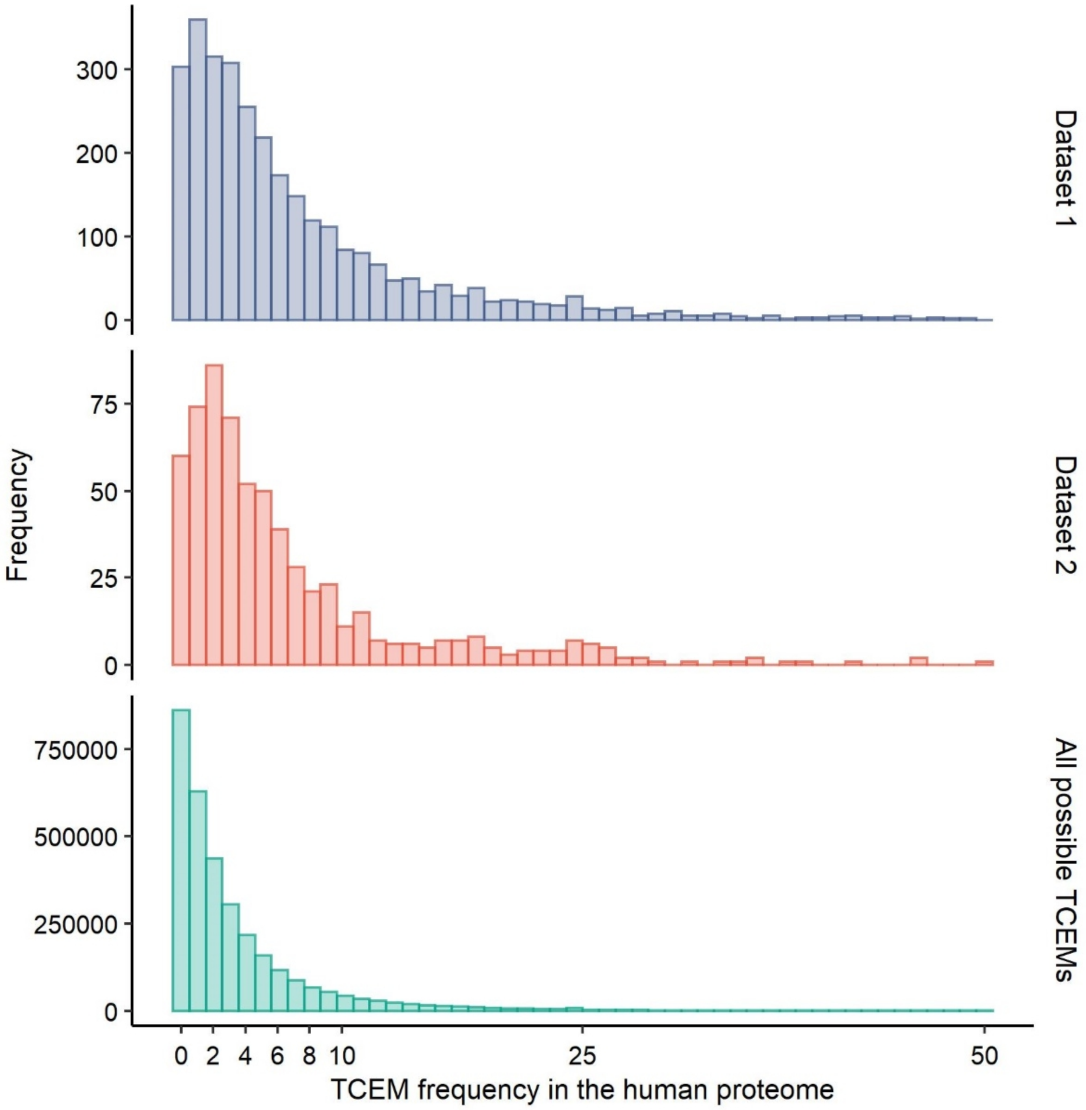

Fig. S3. The distribution of TCEM frequency in the human proteome. The histograms indicate the number of times TCEMs were found in the human proteome (*n* = 3,194,577, 3,031 and 630 for all possible TCEMs and motifs in datasets 1 and 2, respectively. Note, that only TCEM sequences occurring less than 51 times in the human proteome are shown on the plot for visualization purposes).

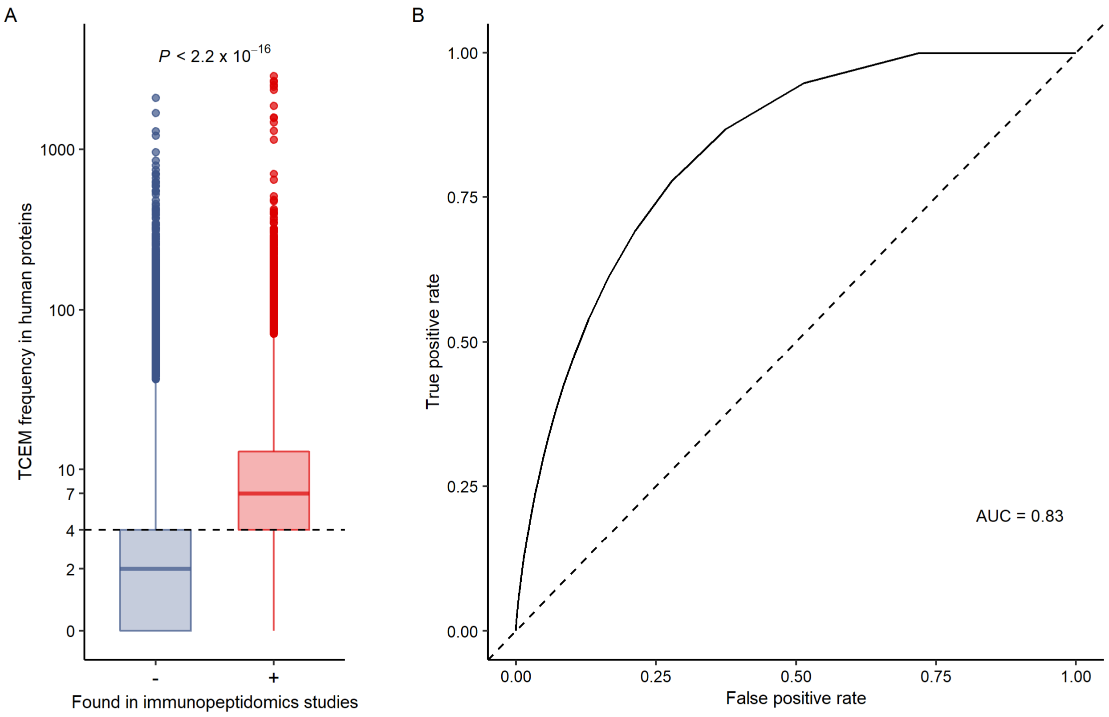

Fig. S4. The frequency of TCEMs in human proteins predicts their presentation by HLA-I molecules on the cell surface. (*A*) TCEM sequences were stratified into two groups based on their prevalence in at least one nine or ten amino acid-long HLA-I-presented peptide identified in immunopeptidomics studies (Dataset S2, *n* = 153,456 and 3,046,544 in identified and nonidentified groups, respectively). The vertical axis indicates the frequency of TCEMs in human proteins. The dashed horizontal line indicates the optimal cutoff for predicting the HLA-presentation of a given TCEM sequence on the cell surface (see the legend of *B* for explanation). The two-sided *P* value of a Wilcoxon's rank-sum test is indicated. (*B*) The ROC curve indicates the accuracy of TCEM frequency in human proteins for predicting their prevalence in at least one HLA-I-presented peptide in immunopeptidomics studies. The optimal cutoff was calculated by selecting the value of TCEM frequency, which minimizes $x^2 + (y-1)^2$, where *x* is the false positive rate, and *y* is the true positive rate. AUC: area under the curve.

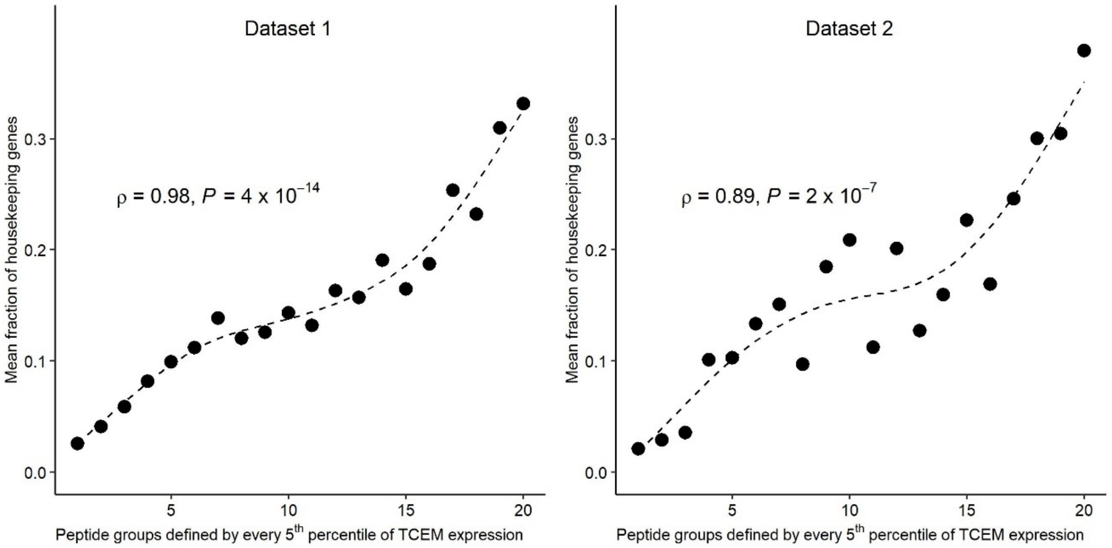

Fig. S5. The immunogenicity of peptides and the prevalence of TCEM-encoding housekeeping genes in different TCEM expression groups. Peptides were classified into twenty groups with increasing TCEM expression in cTECs. Highly expressed TCEMs in cTECs are more likely to be encoded by housekeeping genes. For each TCEM, we collected the genes encoding their sequence. Then, we determined the relative fraction of housekeeping genes among them. The mean of these values is indicated for each TCEM expression group. The dashed lines indicate smooth curve fitted using cubic smoothing spline method in R (*Methods*). Spearman's rho and two-sided correlation test P values are shown.

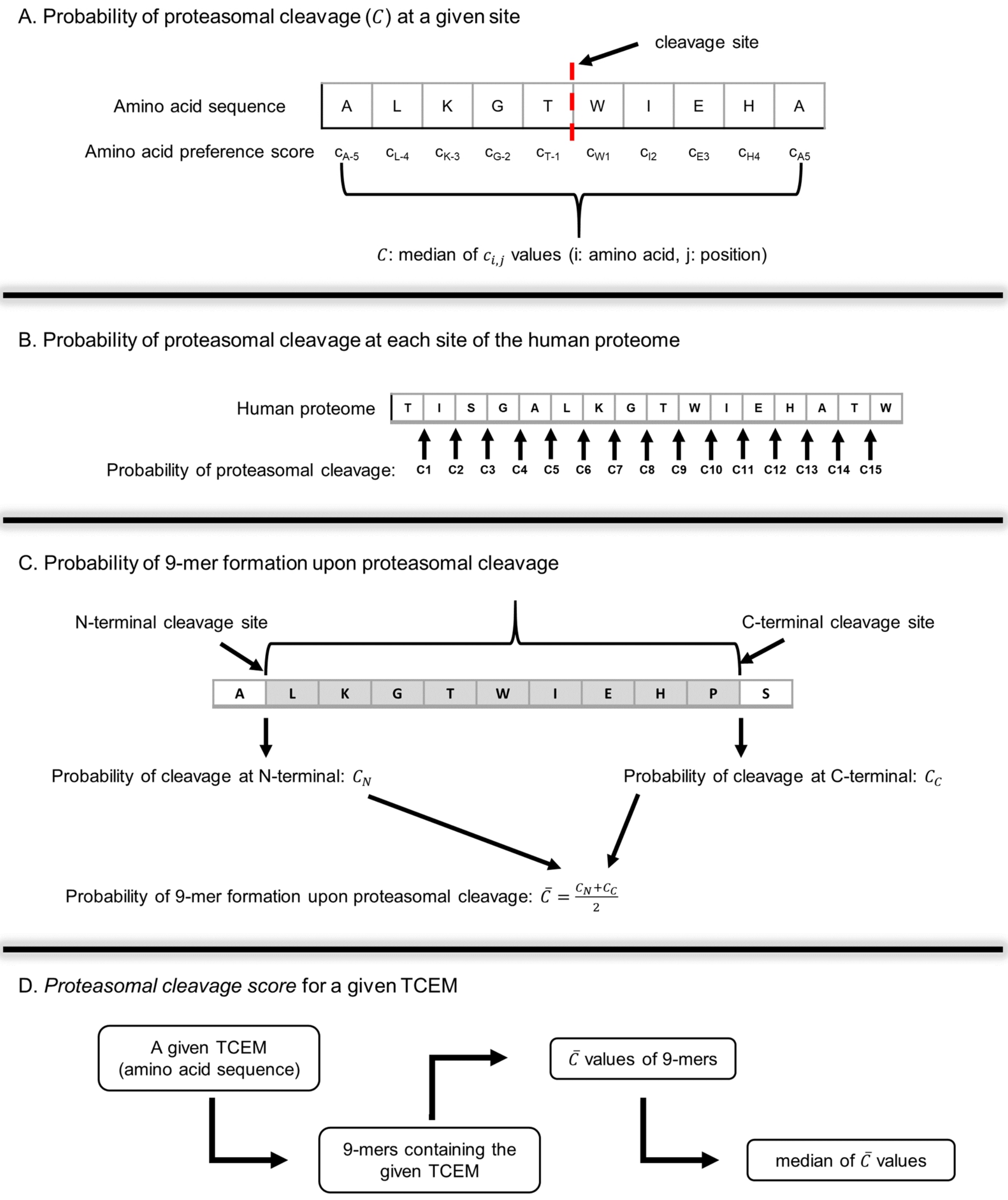

Fig. S6. Calculation of proteasomal cleavage score. (*A*) Amino acid preference values around the proteasomal cleavage site were calculated using data from a previous study (2). The probability of cleavage at a given site of a protein sequence was estimated by calculating the median of preference values associated with the amino acids that were found at the five positions towards the C and N-termini. (*B*) We calculated the probability of proteasomal cleavage ($\boldsymbol{C}$) at each site of the human proteome. (*C*) For each 9-mer in the human proteome, we averaged the $\boldsymbol{C}$ values before the N- and after the C-terminal amino acids to estimate the probability of peptide formation upon proteasomal cleavage ($\bar{\boldsymbol{C}}$). (*D*) Finally, for each TCEM, we calculated the median of $\bar{\boldsymbol{C}}$ values associated with the peptides that include the given TCEM.

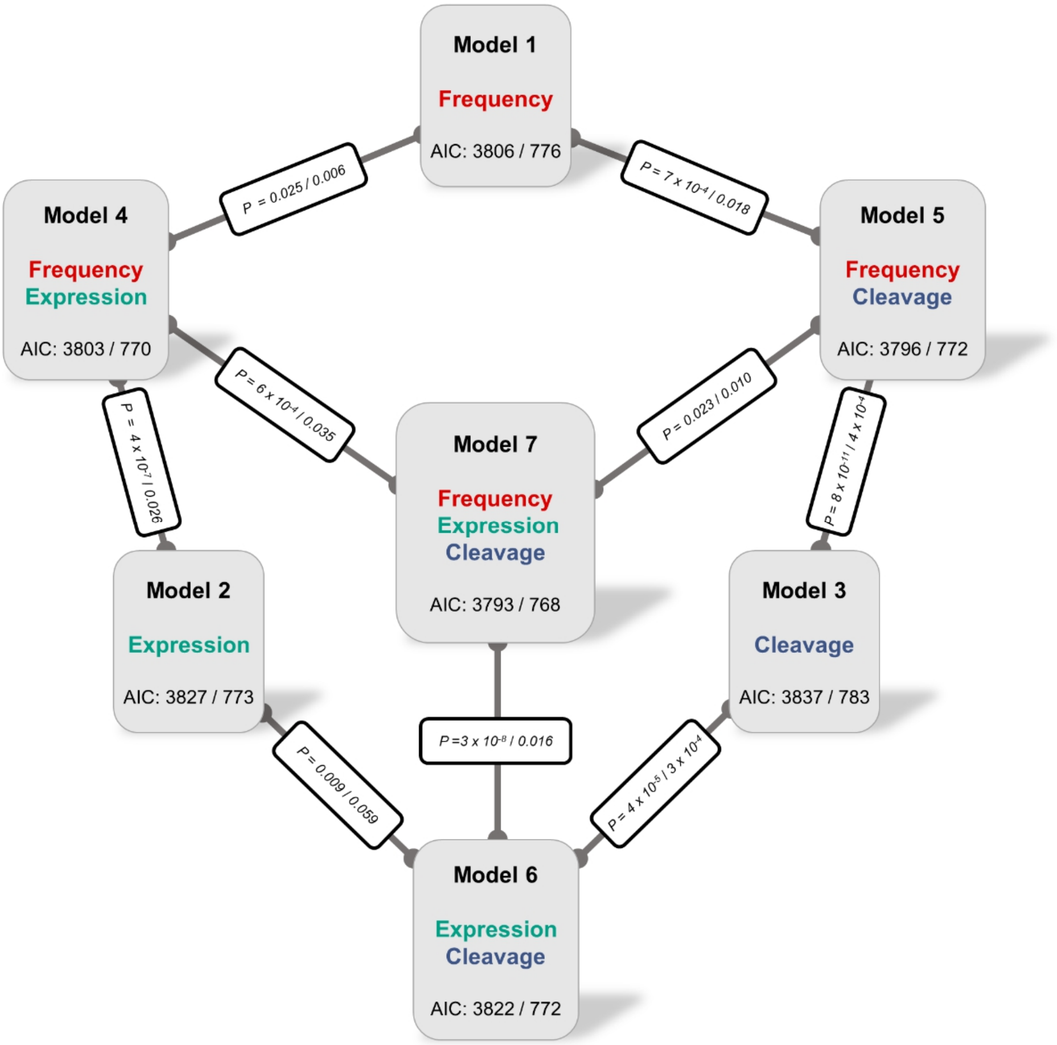

Fig. S7. The effects of TCEM frequency, expression and thymoproteasomal cleavage score are not confounded by and independent of each other. We constructed univariate, bivariate and trivariate logistic regression models to examine the effect of each variable on T cell activation. Models were compared with ANOVA and the two-sided *P* values for datasets 1 (*Left*) and 2 (*Right*) are shown on arrows. *P* values lower than 0.05 indicate that the more complex model fits better than the simpler ones (See also Table S1). Akaike information criterion (AIC) values are shown for datasets 1 (*Left*) and 2 (*Right*). All bivariate models fitted significantly better than univariate ones. Additionally, the trivariate model fitted significantly better than bivariate ones. For detailed data on models, see Table S1.

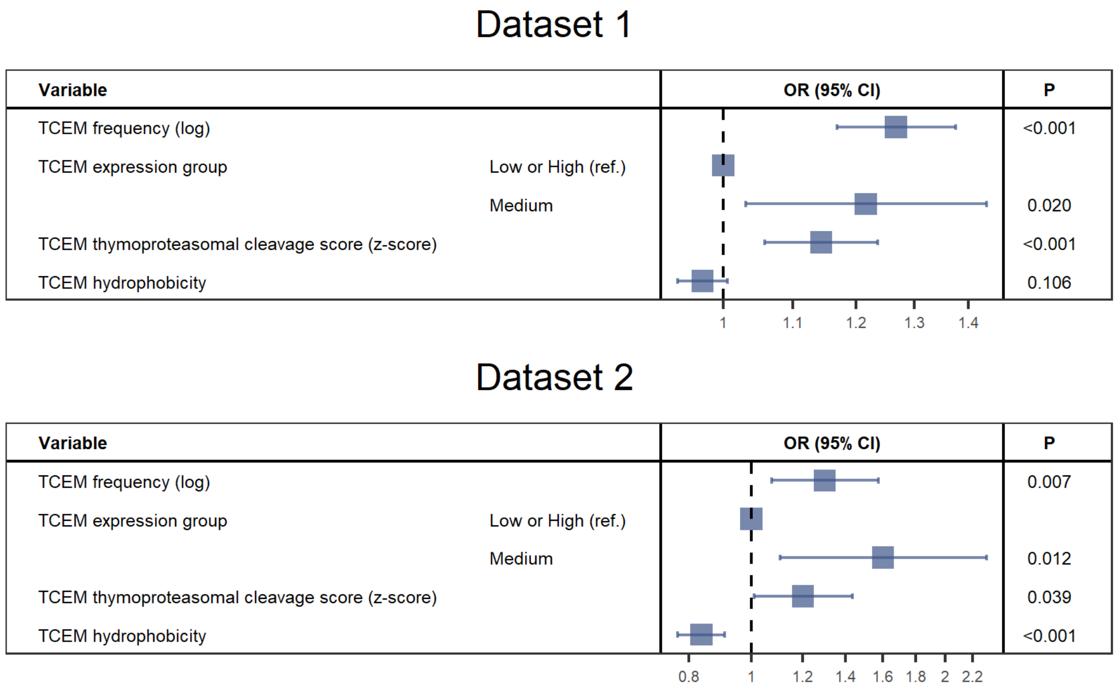

Fig. S8. The effects of TCEM frequency, expression and thymoproteasomal cleavage are not confounded by the hydrophobicity of amino acids. We constructed logistic regression models containing TCEM frequency, thymoproteasomal cleavage score and hydrophobicity as continuous, and TCEM expression as categorical variables. As previously (3), we used the Kyte-Doolittle scale (4) to explain the hydrophobicity of amino acids at the TCEM region. Then, we calculated the median of amino acid-specific values. We log-transformed TCEM frequency and calculated z-score for thymoproteasomal cleavage score values. The OR with 95% confidence interval is indicated. Two-sided *P* values of Z statistics are shown.

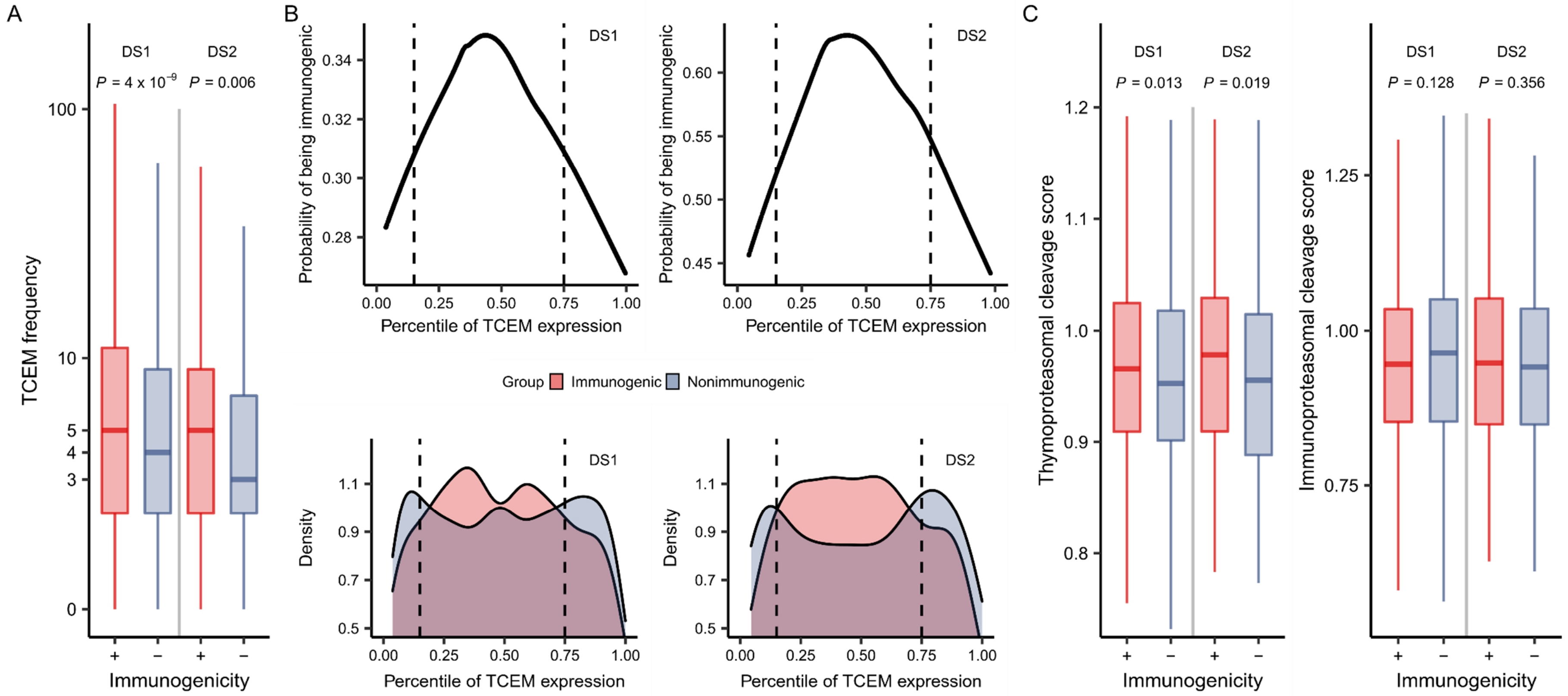

D

	TCEM frequency < 4	TCEM expression < 15%	TCEM expression > 75%	Thymoproteasomal cleavage score < 25%	Immunoproteasomal cleavage score < 25%
Dataset 1 OR (P)	0.68 (9 x 10^{-7})	0.73 (0.008)	0.75 (0.003)	0.79 (0.014)	1.01 (0.96)
Dataset 2 OR (P)	0.63 (0.006)	0.48 (0.003)	0.56 (0.004)	0.68 (0.049)	0.99 (1.00)

Fig. S9. The effects of TCEM frequency, expression and proteasomal cleavage on immunogenicity after excluding peptides that bind to HLA molecules with secondary anchors at their TCEM region. (*A*) The plot indicates the number of times immunogenic (+, *n* = 1,035 and 345 in datasets 1 and 2, respectively) and nonimmunogenic (-, *n* = 2,276 and 274 in datasets 1 and 2, respectively) TCEMs found in human proteins. In both datasets, TCEMs of immunogenic peptides were found more times in human proteins than TCEMs of nonimmunogenic ones. Outliers are not shown for visualization purposes. (*B*) The upper plots show the probability of a TCEM being immunogenic as the function of its expression in cTECs. The curves were fitted using lowess regression (5). The lower plots indicate the probability density of the expression of immunogenic (*n* = 947 and 312 for datasets 1 and 2, respectively) and nonimmunogenic (*n* = 2,029 and 246 for datasets 1 and 2, respectively) TCEMs. For visualization purposes, gene expression values were transformed by calculating their percentile rank. Vertical dashed lines indicate cutoff values used for OR calculation in D. (*C*) The likelihood of TCEM formation after thymoproteasomal (*Left*) and immunoproteasomal (*Right*) cleavage is shown. TCEMs of immunogenic peptides were more likely to be generated and presented after thymoproteasomal, but not immunoproteasomal cleavage. *n* = 947 and 313 for immunogenic and 2,035 and 247 for nonimmunogenic TCEMs in datasets 1 and 2, respectively. Outliers are not shown for visualization purposes. (*D*) Peptides were classified based on their TCEM's frequency in human proteins, expression in cTECs and thymo- or immunoproteasomal cleavage scores. TCEMs found rarely in the human proteome, having low expression in cTECs or low thymoproteasomal cleavage score were less likely to be immunogenic. *P* values of two-sided Fisher's exact tests are shown. In *A* and *C*, the *P* values of two-sided Wilcoxon's rank-sum tests are indicated. On *A* and *C*, the bottom and top of boxes indicate the first and third quartile, horizontal lines indicate median, vertical lines indicate first quartile - 1.5*IQR and third quartile + 1.5*IQR. DS1: dataset 1, DS2: dataset 2.

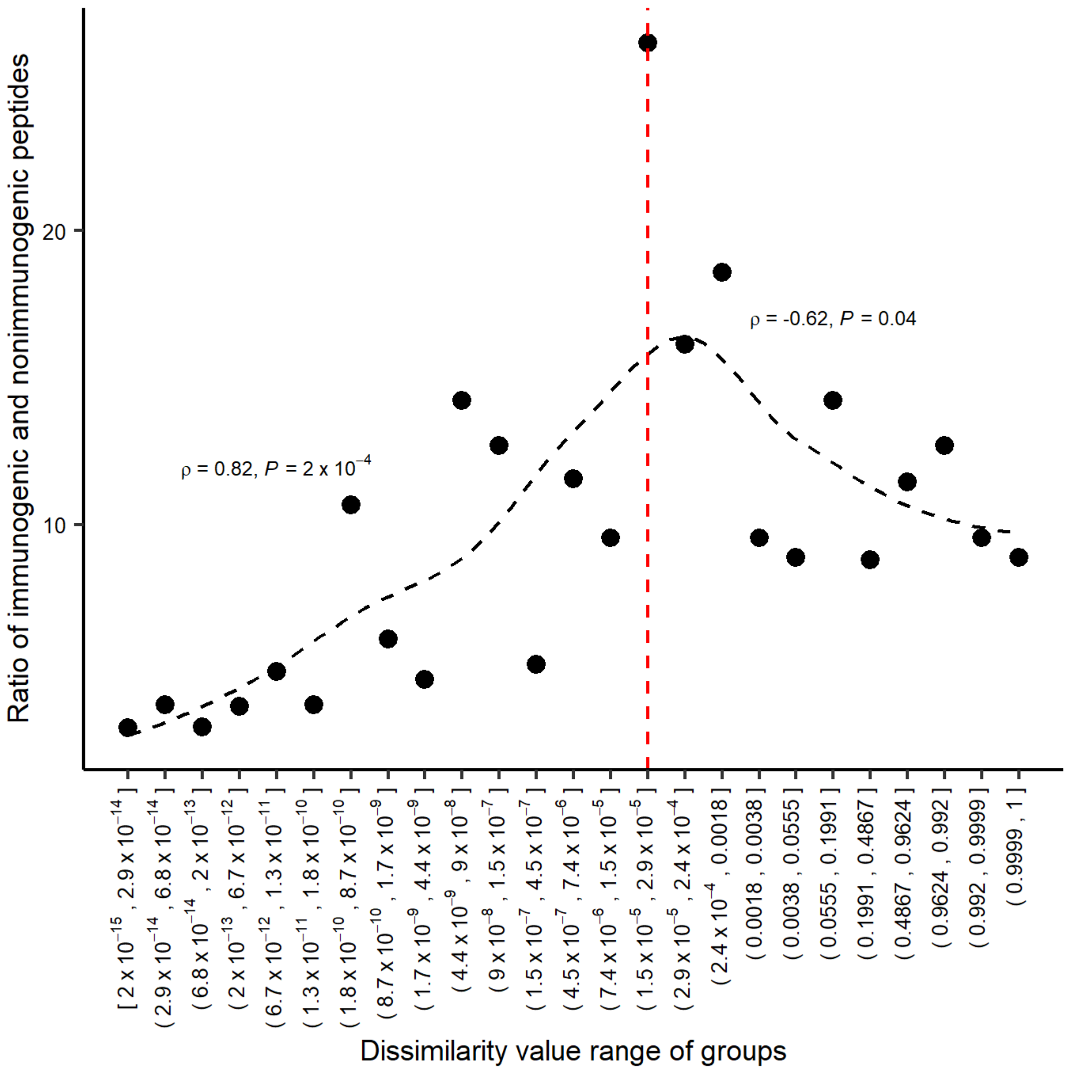

Fig. S10. Peptide dissimilarity to human proteins and immunogenicity. Peptides with dissimilarity values were acquired from a recent study (6) and the ones also found in dataset 1 or 2 were discarded resulting in 3,430 sequences. Peptides were stratified into twenty-five groups based on dissimilarity and the ratio of immunogenic and nonimmunogenic peptides is shown in increasing order of dissimilarity. The red dashed line indicates the group having the highest fraction of immunogenic peptides. Spearman's rho and the corresponding two-sided correlation test P- values are indicated for the ascending and descending part of the plot separately. The black dashed line indicates a smooth curve fitted using a cubic smoothing spline method in R (*Methods*). To note, using BLOSUM62 similarity values (as in Fig. 3B) would have been ill-suited for this analysis because the dataset contains only a very low number of nonimmunogenic peptides. As a consequence, many peptide groups would have contained zero nonimmunogenic peptides. This is not the case when using dissimilarity values of the original study, which could be explained by their different distribution from that of BLOSUM62 similarity values.

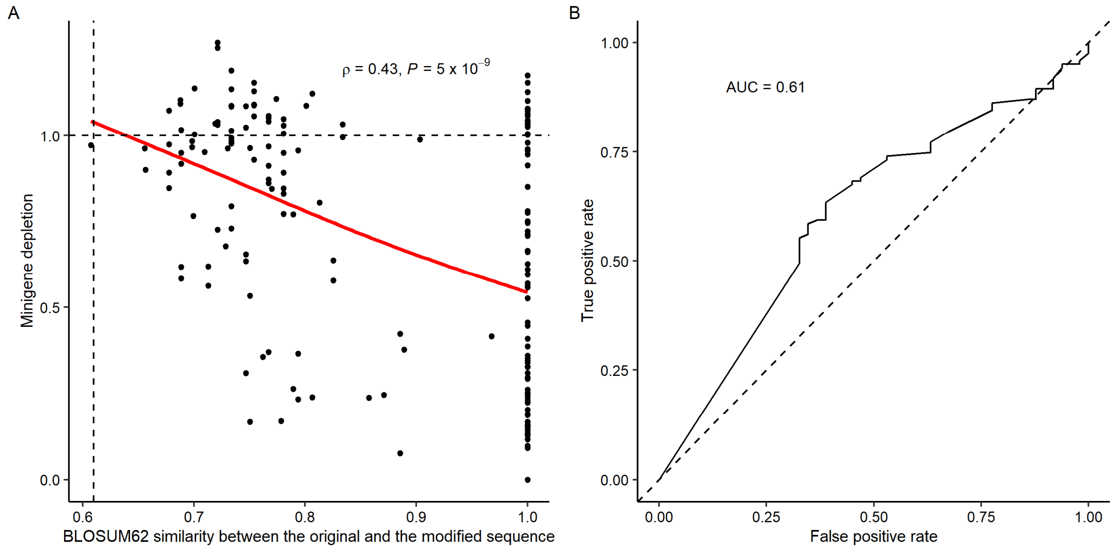

Fig. S11. Cross-reactivity of the A6 TCR. A recent study measured the ability of A6 TCR to bind single amino acid mutants of its target epitope, the Tax peptide of HTLV-1 in a minigene depletion assay (7). (*A*) The relationship between TCEM sequence similarity and TCR binding strength. The vertical axis indicates the frequency of minigenes in A6 cocultured libraries relative to their frequency in the library before co-culture. Smaller values represent higher depletion and, thus, stronger binding by the TCR. TCEM similarity to the original, nonmutated peptide was associated with larger minigene depletion (i.e., stronger binding by TCR). We considered the lack of depletion (value of 1.0 on the vertical axis, marked with a horizontal line) as nonbinding by the TCR. Spearman's rho and a two-sided correlation test *P* value are indicated. The red line indicates a smooth curve fitted using a cubic smoothing spline method in R (*Methods*). *n* = 172. (*B*) The ROC curve is showing the accuracy of BLOSUM62 similarity for classifying peptides into the nonbinding group. The optimal cutoff for classification (0.61) is indicated with a vertical line on *A*.

Table S1. Detailed description of logistic regression models on Fig. S7. The coefficients and the two-sided *P* values of Z statistics are indicated for each independent variable. AIC: Akaike information criterion

Dataset	Model	TCEM frequency coefficient	TCEM frequency *P* value	TCEM expression coefficient	TCEM expression *P* value	TCEM thymoproteasomal cleavage score coefficient	TCEM thymoproteasomal cleavage score *P* value	AIC
Dataset 1	model 1 (univariate)	2.31	2×10^{-9}					3806
Dataset 1	model 2 (univariate)			0.32	8×10^{-5}			3827
Dataset 1	model 3 (univariate)					1.02	0.017	3837
Dataset 1	model 4 (bivariate)	0.20	4×10^{-7}	0.19	0.025			3803
Dataset 1	model 5 (bivariate)	0.25	1×10^{-10}			1.50	7×10^{-4}	3796
Dataset 1	model 6 (bivariate)			0.33	5×10^{-5}	1.13	0.009	3822
Dataset 1	model 7 (trivariate)	0.23	4×10^{-8}	0.19	0.023	1.51	6×10^{-4}	3793
Dataset 2	model 1 (univariate)	0.29	9×10^{-4}					776
Dataset 2	model 2 (univariate)			0.65	2×10^{-4}			773
Dataset 2	model 3 (univariate)					1.96	0.035	783
Dataset 2	model 4 (bivariate)	0.21	0.027	0.51	0.006			770
Dataset 2	model 5 (bivariate)	0.31	5×10^{-4}			2.18	0.020	772
Dataset 2	model 6 (bivariate)			0.63	3×10^{-4}	1.76	0.061	772
Dataset 2	model 7 (trivariate)	0.23	0.017	0.47	0.010	1.97	0.037	768

Table S2. The effect of TCEM attributes on immunogenicity is additive. In both datasets, rare TCEMs having low expression in cTECs and low thymoproteasomal cleavage score were associated with much lower immunogenicity than TCEMs explained by one or two of the three attributes. OR: Odds ratio (immunogenic vs. nonimmunogenic) in the examined TCEM group. Two-sided *P* values of Fisher's exact tests are shown.

Dataset	TCEM frequency < 4	TCEM expression < 15%	TCEM thymoproteasomal cleavage score < 25%	OR	*P* value
Dataset 1	●			0.68	3×10^{-7}
Dataset 1		●		0.71	0.002
Dataset 1			●	0.77	0.004
Dataset 1	●	●		0.44	2×10^{-6}
Dataset 1	●		●	0.64	0.003
Dataset 1		●	●	0.50	0.002
Dataset 1	●	●	●	0.31	**0.002**
Dataset 2	●			0.61	0.003
Dataset 2		●		0.48	0.003
Dataset 2			●	0.67	0.041
Dataset 2	●	●		0.43	0.009
Dataset 2	●		●	0.47	0.007
Dataset 2		●	●	0.33	0.018
Dataset 2	●	●	●	0.26	**0.027**

Table S3. The results remained when TCEMs containing certain amino acids were excluded from the analysis. In the third, seventh, eighth, ninth and tenth columns, odds ratio (OR) values and two-sided *P* values of Fisher's exact tests are indicated. In the sixth column, two-sided *P* values of Wilcoxon's rank-sum tests are shown.

Full name and one letter code of amino acids		Prevalence in human proteins*	Fisher's exact test OR (*P*) - Amino acid prevalence in IMM vs. NIMM TCEMs	Median TCEM freq. in IMM	Median TCEM freq. in NIMM	Wilcoxon's rank-sum test *P* - TCEM freq. in IMM vs. NIMM (Fig. 1A)	Fisher's exact test OR (*P*) - low vs. medium TCEM expression (Fig. 1D)	Fisher's exact test OR (*P*) - high vs. medium TCEM expression (Fig. 1D)	Fisher's exact test OR (*P*) - low vs. high thymoprot. cleavage score (Fig. 1D)	Fisher's exact test OR (*P*) - low vs. high immunoprot. cleavage score (Fig. 1D)
Tyrosine	Y	0.027	0.46 (4×10^{-28})	6	5	8×10^{-4}	0.75 (0.011)	0.73 (6×10^{-4})	0.70 (5×10^{-5})	1.08 (0.400)
Alanine	A	0.070	1.69 (5×10^{-19})	5	4	0.001	0.66 (9×10^{-4})	0.81 (0.036)	0.79 (0.017)	0.88 (0.181)
Glycine	G	0.066	1.77 (2×10^{-18})	5	4	9×10^{-6}	0.63 (1×10^{-4})	0.75 (0.003)	0.74 (0.001)	0.92 (0.373)
Phenylalanine	F	0.036	0.63 (5×10^{-15})	6	5	1×10^{-6}	0.68 (0.001)	0.73 (0.001)	0.81 (0.024)	1.03 (0.778)
Isoleucine	I	0.043	0.65 (1×10^{-14})	6	4	7×10^{-9}	0.67 (0.001)	0.66 (4×10^{-5})	0.82 (0.040)	1.01 (0.961)
Glutamic acid	E	0.071	1.61 (2×10^{-10})	5	4	1×10^{-7}	0.72 (0.004)	0.69 (9×10^{-5})	0.74 (9×10^{-4})	1.08 (0.374)
Proline	P	0.063	1.52 (6×10^{-10})	5	4	1×10^{-7}	0.66 (4×10^{-4})	0.71 (2×10^{-4})	0.79 (0.010)	1.06 (0.555)
Tryptophan	W	0.012	1.95 (6×10^{-9})	6	4	1×10^{-14}	0.64 (2×10^{-5})	0.70 (5×10^{-5})	0.74 (5×10^{-4})	1.06 (0.458)
Serine	S	0.083	0.71 (6×10^{-9})	4	3	6×10^{-9}	0.66 (8×10^{-4})	0.67 (6×10^{-5})	0.83 (0.055)	1.05 (0.597)
Lysine	K	0.057	0.64 (4×10^{-8})	5	4	4×10^{-8}	0.65 (1×10^{-4})	0.73 (6×10^{-4})	0.79 (0.008)	0.99 (0.896)
Valine	V	0.060	1.25 (2×10^{-4})	5	4	2×10^{-6}	0.70 (0.003)	0.76 (0.006)	0.82 (0.045)	1.04 (0.703)
Asparagine	N	0.036	0.82 (0.004)	5	4	2×10^{-7}	0.63 (6×10^{-5})	0.75 (0.002)	0.71 (2×10^{-4})	1.00 (1.000)
Threonine	T	0.054	1.19 (0.004)	5	4	2×10^{-11}	0.77 (0.029)	0.64 (1×10^{-5})	0.65 (2×10^{-5})	1.02 (0.886)
Glutamine	Q	0.048	1.28 (0.013)	5	4	3×10^{-8}	0.69 (5×10^{-4})	0.72 (2×10^{-4})	0.78 (0.005)	1.05 (0.584)
Aspartic acid	D	0.047	1.18 (0.031)	5	4	2×10^{-6}	0.76 (0.0153)	0.74 (0.001)	0.77 (0.004)	1.03 (0.756)
Arginine	R	0.056	1.15 (0.065)	5	4	2×10^{-8}	0.64 (7×10^{-5})	0.75 (0.002)	0.72 (2×10^{-4})	1.00 (1.000)
Methionine	M	0.021	0.85 (0.091)	6	5	3×10^{-8}	0.72 (0.002)	0.75 (9×10^{-4})	0.75 (8×10^{-4})	1.04 (0.614)
Histidine	H	0.026	0.89 (0.276)	5	4	4×10^{-9}	0.65 (8×10^{-5})	0.74 (7×10^{-4})	0.74 (3×10^{-4})	1.09 (0.316)
Cysteine	C	0.023	0.91 (0.359)	5	4	2×10^{-9}	0.68 (2×10^{-4})	0.70 (4×10^{-5})	0.72 (1×10^{-4})	1.05 (0.589)
Leucine	L	0.100	1.01 (0.892)	3	3	1×10^{-5}	0.53 (2×10^{-5})	0.77 (0.025)	0.76 (0.018)	1.05 (0.695)

IMM: immunogenic, NIMM: nonimmunogenic; *Selenocysteine (U): 3.17×10^{-6}

Dataset S1 (separate file). All numerical data of T cell activation datasets used in the study.

Dataset S2 (separate file). Data of immunopeptidomics studies.

Dataset S3 (separate file). Numerical data of Fig. 5B, proteome IDs and full names of pathogens.

SI References

1. N. Xiao, D.-S. Cao, M.-F. Zhu, Q.-S. Xu, protr/ProtrWeb: R package and web server for generating various numerical representation schemes of protein sequences. *Bioinformatics* **31**, 1857–1859 (2015).
2. K. Sasaki, *et al.*, Thymoproteasomes produce unique peptide motifs for positive selection of CD8 + T cells. *Nature Communications* **6**, 7484 (2015).
3. D. Chowell, *et al.*, TCR contact residue hydrophobicity is a hallmark of immunogenic CD8+ T cell epitopes. *PNAS* **112**, E1754–E1762 (2015).
4. J. Kyte, R. F. Doolittle, A simple method for displaying the hydropathic character of a protein. *J Mol Biol* **157**, 105–132 (1982).
5. R. Andersen, Nonparametric Methods for Modeling Nonlinearity in Regression Analysis. *Annual Review of Sociology* **35**, 67–85 (2009).
6. L. P. Richman, R. H. Vonderheide, A. J. Rech, Neoantigen Dissimilarity to the Self-Proteome Predicts Immunogenicity and Response to Immune Checkpoint Blockade. *Cell Systems* **9**, 375-382.e4 (2019).
7. R. S. Gejman, *et al.*, Identification of the Targets of T-cell Receptor Therapeutic Agents and Cells by Use of a High-Throughput Genetic Platform. *Cancer Immunol Res* **8**, 672–684 (2020).
